# Methionine epimerization in cyclic peptides†

**DOI:** 10.1039/d1ra04260b

**Published:** 2021-06-11

**Authors:** Pramodkumar D. Jadhav, Jianheng Shen, Peta-Gaye Burnett, Jian Yang, Ramaswami Sammynaiken, Martin J. T. Reaney

**Affiliations:** Department of Plant Sciences, University of Saskatchewan Saskatoon SK S7N 5A8 Canada pramodkumar.jadhav@usask.ca martin.reaney@usask.ca; Drug Discovery and Development Research Group, College of Pharmacy and Nutrition, University of Saskatchewan 107 Wiggins Road Saskatoon SK S7N 5E5 Canada; Saskatchewan Structural Sciences Centre, University of Saskatchewan 110 Science Place Saskatoon SK S7N 5C9 Canada; Prairie Tide Diversified Inc. 102 Melville Street Saskatoon Saskatchewan S7J 0R1 Canada; Guangdong Saskatchewan Oilseed Joint Laboratory, Department of Food Science and Engineering, Jinan University 601, Huangpu Avenue West Guangzhou Guangdong 510632 China

## Abstract

Bioactive flax cyclic octa- and nona-peptides containing single methionine (Met) and its oxidized forms were treated under mild alkaline conditions to perform regio-selective epimerization. Cyclic peptide epimerization at the Met α-proton in a single chemical step has not been reported previously. The epimerization rate varies among Met oxidation states and ring size. These d-amino isomers along with the developed Met alkylation strategy will enable an approach to novel chemical functionalization of biomolecules. The amino acid configurations were confirmed by Marfey derivatizations, and cytotoxicity studies show the difference among the isomers. These d-amino analogs can act as a potential biomarker in plant protein processing and biomedical applications.

## Abstract 2

One step regioselective methionine epimerization in cyclic peptides followed by selective functionalization leads to chemical novelty.

## Graphical abstract

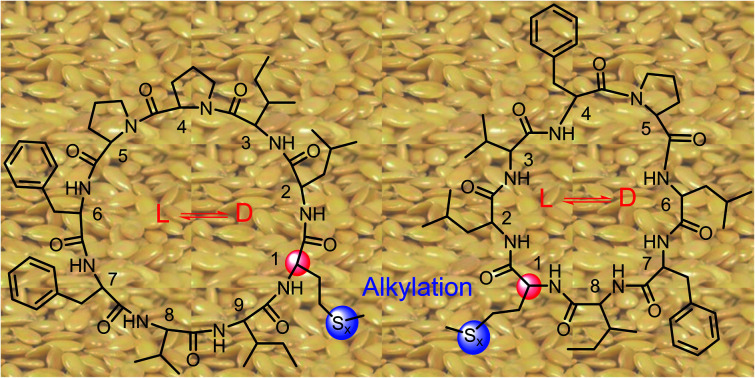

## 1. Introduction

Cyclic peptides belong to an important class of naturally occurring biologically active compounds. For example, tyrocidine A^1^ and gramicidin S^2^ are used as antibiotics whereas cyclosporine A^3^ is used as an immunosuppressant drug. Cyclic peptides are more stable than linear peptides and resist protease degradation, and cyclic Gαi binding peptide [*NaC*-(MITWYEFVAGTK)] has shown proteolytic stability compared to the parent linear molecule.^4^ Furthermore, the presence of d-amino acids enhances structural/conformational flexibility as compared to the l-isomers. Incorporation of d-amino acids can increase biological activity by stabilizing specific molecular conformations. For example, cyclopentapeptide, astin-C analogs containing d-amino acid residues show a better immunosuppressive activity than its isomers.^5^ In the case of omega-agatoxins IVB and IVC, the peptide with d-serine in position 46 is about four times more potent than the l-serine isomer in its inhibitory action on P-type Ca channels in rat cerebellar Purkinje cells.^6^ Another interesting property of d-amino acid peptides is improved resistance to protease hydrolysis that leads to greater stability and improved therapeutic applications. For example, the incorporation of single d-amino acid in a cyclic decapeptide increases stability and maintains breast cancer cell targeting.^7^

Selective transformation of functional groups on biomolecules such as carbohydrates and peptides is important in biomedical research to prepare adducts and study structure–function relationships. Post-translational epimerization of selective amino acids in the peptide chain is usually a formidable challenge and requires enzymes with discriminating competence.^8^ Some examples include *S*-adenosyl-l-methionine radical epimerase family catalyzes l to d-amino acids into ribosomally synthesized peptides.^9–11^ Enzymes have been used to generate d-alanine from l-serine through a two-step process of the dehydration–hydrogenation process.^12^ Also, a peptide epimerase, BotH, helps isomerization of l-Asp to d-Asp during bottromycin biosynthesis.^13^ However, the use of chemical synthesis for selective epimerization on cyclic peptides is very rare. A selective epimerization at Leu of the cyclic depsiheptapeptide *via* 5-aminooxazole intermediate was performed in four synthetic steps.^14^ Another example includes epimerization of bicyclic hexapeptide RA-VII *via* oxazole intermediate.^15^ Classically, incorporation of d-amino acids into peptides has been performed by solid-phase synthesis, where amino acids are sequentially added to solid phase support to prepare linear peptides, and then release them by a reaction that results in cyclic products (*e.g.* Merrifield resin). These technologies consist of specialized resin, hazardous solvents, reagents, and intensive chromatography that can lower the yield of a peptide synthesis.^5,16–18^ In addition, site-directed chemical modification without affecting other functional groups is often a formidable challenge. Cyclic peptides provide a unique opportunity for selective functionalization of identical functional groups. For example, vitamin B12 biomimetic synthesis, the nucleotide component selectively reacts with only one ester group of cobyrinic acid heptakis(cyanomethylester).^19,20^

Plant cyclic peptides lacking disulfide bonds with head-to-tail cyclization from proteinogenic amino acids were termed orbitides.^21^ These generally contain amino acids in l-form with exceptions including, schnabepeptide (cyclooctapeptide) found in *Schnabelia oligophylla* containing d-Trp.^22^ Flaxseed orbitide LO 1 (cyclolinopeptide A), first discovered in a precipitated flaxseed oil slime, possesses immunosuppressive activity (ESI, Table S1†).^23,24^ About 39 flax orbitides containing proteinogenic amino acids (such as Gly, Pro, Phe, Val, Leu, Ile, Met, and Trp) were reported and have various biological activities including acting as immunosuppressants, antioxidants, anti-inflammatories, anticancer agents and osteoclast differentiation inhibitors.^25^ The aim of the study is to exploit conformational differentiation in a cyclic peptide system for selective Met epimerization. The Met containing orbitides are shown in Table S1 (ESI†).

**Scheme 1:**
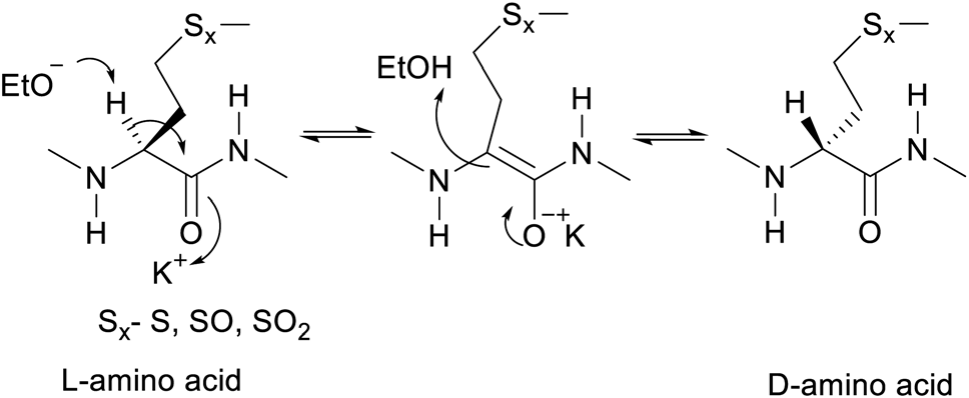
Proposed mechanism of base catalyzed epimerization.

**Fig. 1:**
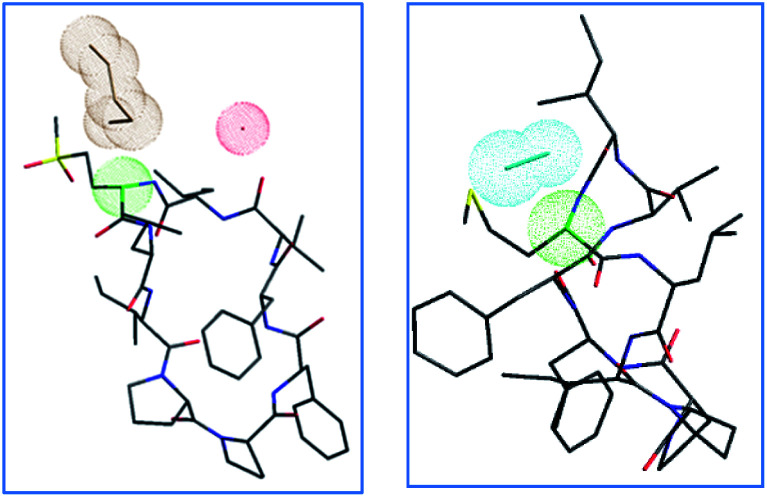
Crystal structures of LO 4 butanol solvate^29^ and LO 2 methanol solvate,^30^ with color-coded atoms sulfur – yellow, oxygen – red, nitrogen – blue, carbon – black, α carbon Met/MetO_2_ – green, solvent clouds: butanol – brown, water – red, methanol – cyan.

The proposed mechanism of epimerization is shown below in Scheme 1. Generally, racemases and epimerases are enzymes that catalyze the epimerization of biological molecules. Enzymes utilize a one- or two-step mechanism to subtract the α-H proton of an amino acid in a protein or peptide by a deprotonation/reprotonation mechanism.^8,26^ In the current study, the base (potassium ethoxide) abstracts protons from one face and solvent protonation occurs through another face to produce a d-amino isomer. The acidity of α-H of different amino acid residues such as Leu, Ile, Phe, Val, and Met are quite similar^27,28^ and hence the Met epimerization was not considered kinetically favored over other amino acids in this cyclic system. To afford selective epimerization at the Met site there might be two possible mechanisms. The orbitides cyclic structure imposes a steric constraint allowing base to have selective access to α-H of Met or there is a polar effect of sulfur, where solvents such as butanol and methanol were bonded close to MetO_2_ and Met in LOs 4 and 2 respectively. This is observed in the crystal structure as shown in Fig. 1.^29,30^ Hence solvent can play a major role in resonance stabilization of the carbanion intermediate generated by base thus allowing base access for proton abstraction. Also, there were reports of oxazolone mechanism to support the effect of sidechain on Met epimerization. The proximity of a Lewis base in the structure of Met and its oxidized products might stabilize isomerization intermediates.^31,32^ We have found experimentally that eight-membered rings were more prone to epimerization as compare to nine membered (Table 1). Hence steric factors and ring orientation potentially affect alpha proton abstraction.

**Table 1:** % l to d conversion table

LO	Area under curve	LO	Area under curve	% l to d conversion
2	574.834	8	31.1244	5.14
3	2314.17	9	964.877	29.43
4	1251.1	10	322.223	20.48
5	767.894	11	227.048	22.82
6	411.138	12	508.19	55.28
7	319.425	13	502.883	61.16

## 2. Materials and methods

### 2.1 Reagents

d-Isomer of Met LOs 8, 9, 10, and 12 were custom synthesized by Chempeptide Limited (Shanghai, China) and other chemicals were obtained from Sigma-Aldrich Canada Ltd., Oakville, ON, Canada. HPLC grade solvents were used for LC and MS analysis. Prairie Tide Diversified Inc. (Saskatoon, SK Canada) supplied LOs for the study. LOs stock solutions for cytotoxicity assay were prepared in dimethyl sulfoxide (DMSO). Human breast cancer triple-negative-subtype cell line MDA-MB-231 and HER2-subtype cell line Sk-Br-3 were obtained from American Type Culture Collection (ATCC, Manassas, VA, USA). McCoy's 5A Modified Medium, Leibovitz's L-15 Medium and cell culture media, were obtained from Thermo Fisher Scientific (Ottawa, ON, Canada). CytoTox Green Cytotoxicity Assay was acquired from the Promega Corporation (Madison, WI, USA).

### 2.2 Cell culture

A Forma Series II 3110 Water-Jacketed CO_2_ Incubator (Thermo Fisher Scientific) containing T-75 cell culture flasks at 37 °C under a humidified atmosphere was used to culture human breast cancer cell lines MDA-MB-231 and Sk-Br-3. 10% fetal bovine serum (FBS) and 1% penicillin under 5% CO_2_ were used to supplement McCoy's 5A Modified Medium to culture Sk-Br-3 cell line, and 10% FBS and 1% penicillin under 0% CO_2_ were used to supplement Leibovitz's L-15 Medium to culture MDA-MB-231 cell line. Each cell line's culture medium was changed every 2–3 days.

### 2.3 Instrumentation

#### 2.3.1 Liquid chromatography methods

HPLC data was collected at a wavelength range of 190–300 nm using Agilent 1200 series HPLC system equipped with an autosampler, quaternary pump, diode array detector and degasser. Chromatographic separations for Marfey's derivatives were conducted on 100 mm × 4.6 mm i.d., reversed-phase Chromolith® Performance RP-18e columns (Merck KGaA, Darmstadt, Germany). The mobile phase consisted of H_2_O and 0.05 M triethyl phosphate buffer at a flow rate of 2 mL min^−1^ (85 : 15 for 3 min, 85 : 15 to 60 : 40 in 18 min, to 10 : 90 in 0.5 min, 10 : 90 for 1 min, to 85 : 15 in 0.5 min, to equilibration for 2 min) with column compartment at 30 °C. Chromolith® High Resolution RP-18e columns 50 mm × 4.6 mm i.d. (Merck KGaA, Darmstadt, Germany) equipped with in-line filters were used for LO analytical characterization. H_2_O and CH_3_CN linear HPLC gradient with a flow rate of 2 mL min^−1^ (50 : 50 to 20 : 80 for 1.8 min, 20 : 80 to 10 : 90 in 0.1 min, to 50 : 50 in 0.1 min, to equilibration for 1 min), and a column compartment at 32 °C was used.

Preparative reversed phase chromatography for separation of natural and KOH treated LOs 4 and 7 was performed using Chromolith® High Resolution RP-18e column (100 × 4.6 mm i.d., Merck KGaA, Darmstadt, Germany) at 214 nm. The mobile phase consisted of H_2_O–CH_3_CN (50 : 50 for 6.5 min at 1.2 mL min^−1^, 50 : 50 to 5 : 95 in 0.25 min at 1.2 mL min^−1^, 5 : 95 for 0.5 min at 2 mL min^−1^, 5 : 95 to 50 : 50 in 0.25 min at 2 mL min^−1^, to equilibration for 2.5 min at a flow rate of 2 mL min^−1^) with column compartment at 32 °C. Separation of natural and KOH treated LOs 3 and 6 was performed with same column. The mobile phase consisted of H_2_O–CH_3_CN (50 : 50 for 7.0 min at 0.8 mL min^−1^, 50 : 50 to 10 : 90 in 0.25 min at 0.8 mL min^−1^, 10 : 90 to 50 : 50 in 0.25 min at 2 mL min^−1^, to equilibration for 2.5 min at a flow rate of 2 mL min^−1^) with the column compartment at 32 °C.

#### 2.3.2 Mass spectrometric analyses

MicrOTOF-Q II hybrid quadrupole time of flight (Bruker Daltonik GmbH, Bremen, Germany) with Apollo II electrospray ionization (ESI) ion source connected with Agilent 1200 series HPLC system was used for LCMS and LC MS/MS analysis. The MS instrument was operated at a drying gas temperature held at 200 °C, nebulizer gas at 4.0 bar and capillary voltage of −4500 V with Chromolith® FastGradient RP-18e column (50 mm × 2.0 mm i.d., Merck KGaA, Darmstadt, Germany). A gradient at a flow of 0.40 mL min^−1^ of 0.1% formic acid in H_2_O and 0.1% formic acid in CH_3_CN (60 : 40 for 2 min, to 10 : 90 in 8 min, to 60 : 40 in 0.5 min, to equilibration for 5.5 min) was used for chromatography.

#### 2.3.3 Nuclear magnetic resonance

Proton NMR spectra were recorded on a 600 MHz Bruker Avance spectrometer (5 mm PABBO BB-probe head; TopSpin 3.2 Software). The ^1^H NMR spectra chemical shift (*δ*) values are reported in parts per million (ppm) relative to the internal standard TMS. NMR experiments were performed at 298 K using mixture of 70% CD_3_OD and 30% D_2_O as deuterated solvent for kinetic studies. 25 mg LOs 3 and 6 mixtures were dissolved in 600 μL deuterated solvent, transferred to 5 mm NMR tube for ^1^H NMR experiments. The homogeneity of the magnetic field was adjusted by gradient shimming on the *z*-axis and the probe had been tuned and matched in the NMR spectrometer. To study kinetics 30 μL of KOH solution (6.25 mg KOH in deuterated solvent) was added to LO containing NMR tubes, which were then vortexed for 5 s and immediately placed into the NMR spectrometer. Multiple acquisitions were performed with 16 scans and 10 s delays between scans without removal of the tube from the NMR spectrometer until 50 experiments were performed with total acquisition time of 64.1 min.

### 2.4 Synthesis

#### 2.4.1 Alkaline treatment of LOs

LO (1 g) was dissolved in 70% aqueous ethanol (2.5 mL) and KOH (0.25 g) was added. The mixture was sonicated for 30 min. A portion of the mixture (∼1 mL) was transferred to a glass vial and extracted with EtOAc (∼3 mL) then settled. The upper EtOAc phase was transferred by pipette to a clean tube and dried under airflow. This was reconstituted in MeOH for HPLC analysis.

#### 2.4.2 Hydrolysis of cyclic peptide

Cyclic peptides were hydrolyzed in a sealed tube using 6 M HCl at 110 °C for 17 h under argon at a concentration of 1 mg mL^−1^. Concentrated HCl was evaporated using a rotary evaporator to obtain the amino acids.

#### 2.4.3 Marfey derivatization

Amino acids were individually modified by mixing 10 μL of the amino acid (100 mM) with 20 μL of NaHCO_3_ (1 M) and 40 μL H_2_O followed by 90 μL (1% Marfey in acetone). The mixture was heated at 40 °C for 90 min. The reaction was stopped by addition of 20 μL 1 M HCl and the mixture was diluted with 200 μL CH_3_CN. Finally, water was added to a final volume of 1 mL and subjected to HPLC analysis.

### 2.5 Cytotoxicity assay

MDA-MB-231 and Sk-Br-3 cells were plated in 96-well plates at 8000 cells per well (final culture volume – 300 μL) and allowed to grow overnight for attachment before being treated with LO. LO stock solutions were prepared in DMSO at various concentrations of 200 μM; 100 μM; 50 μM, 25 μM, 12.5 μM. CytoTox Green reagent was added to wells during treatments. DMSO treated cells (final concentration at 2%) were used as a negative control. Following the manufacturer's recommended protocols, LOs cytotoxicity was continuously monitored at 24 h intervals for 120 h and calculated using the equation.Cytotoxicity (%) = [OD (experiment) − OD (vehicle control)]/[OD (maximum cell death) − OD (vehicle control)] × 100%

### 2.6 Statistical analysis

Statistical analyses of the results were performed using GraphPad Prism 6 (GraphPad Software, La Jolla, CA, USA) using a one-way ANOVA. Significance differences are indicated by asterisks: *p ≤ 0.05, **p ≤ 0.01, ***p ≤ 0.001, and ****p ≤ 0.0001.

## 3. Results and discussion

**Scheme 2:**
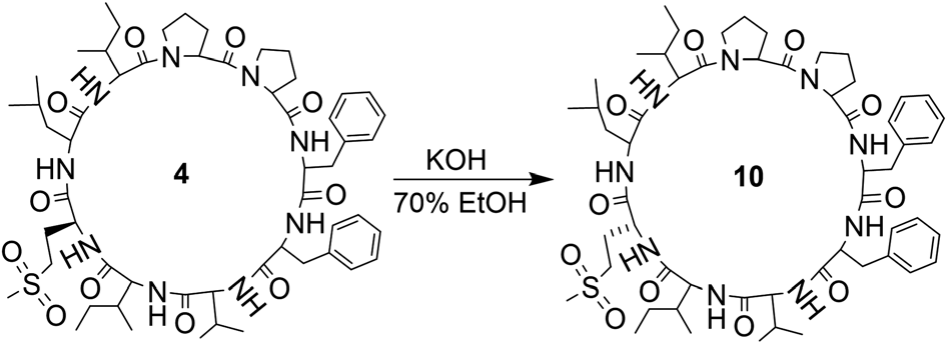
Alkaline treatment of LO 4.

**Fig. 2:**
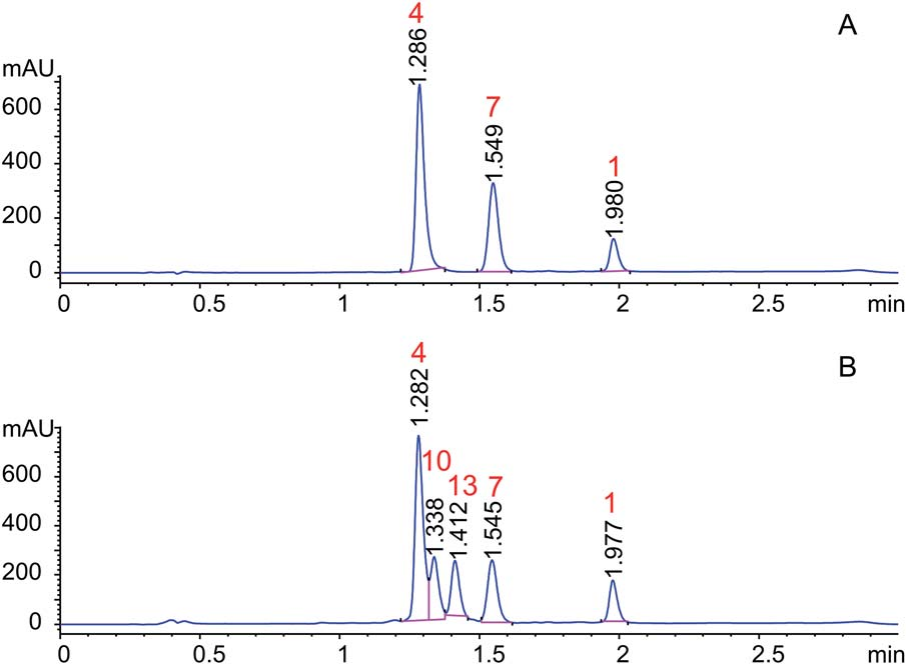
HPLC chromatogram of (A) LOs 1, 4 and 7; (B) KOH treated LOs 1, 4 and 7.

Orbitides containing one Met or their oxidized forms with eight or nine amino acids in the chain were selected. l-forms were naturally available from flax (isolated) while the d-forms were chemically synthesized. These cyclic peptides contain other hydrophobic amino acids such as Pro, Phe, Val, Leu, and Ile. Initially, LOs 1, 4 and 7 mixtures treated with KOH in 70% aqueous ethanol (Scheme 2). But we observed two additional peaks in the HPLC chromatogram at 214 nm and 280 nm (Fig. 2). It suggests that there is a change in the structure of these peptides. Hence MS/MS of these samples was performed to characterize the new compounds and similar molecular masses of the new peaks like 4 and 7 and similar fragmentation patterns were observed. However, no extra peak was observed for non-Met containing LO 1. Hence, this suggests that amino acid isomers might have formed to give the same molecular mass. The LO 4 (l-MetO_2_) was compared with synthetic analog LO 10 (d-MetO_2_), as these LO analogs are diastereomers in the chiral environment, they were resolved in reversed phase HPLC with LO 4 elutes first and KOH treated LO 4 shows an additional peak (Fig. 2). We performed the NMR study to monitor alkaline treatment of LOs 3 and 6 mixture with multiple acquisitions and time delay experiment. 50 NMR acquisitions were performed on a single sample with 16 scans and 10 s delays between each acquisition (total time: 64.1 min). The epimerization reaction happens quickly with few differences in the spectra. Spectral changes were observed on the MetO methyl residues (2.70 ppm) (ESI, Fig. S21†).

To understand the alkaline treatment effect, we must determine all amino acid configurations present in the cyclic peptide. Hence these new peaks were purified using preparative LC techniques. Finally, isolated new cyclic peptides were hydrolyzed and Marfey's derivatives of the amino acids were synthesized to determine amino acid chirality. Marfey's reagent has been used to determine the optical purity of amino acids and peptides. This strategy involves the treatment of racemic analytes with an optical reagent to prepare pairs of diastereomers. These diastereomers were separated using reversed phase chromatography.

**Fig. 3:**
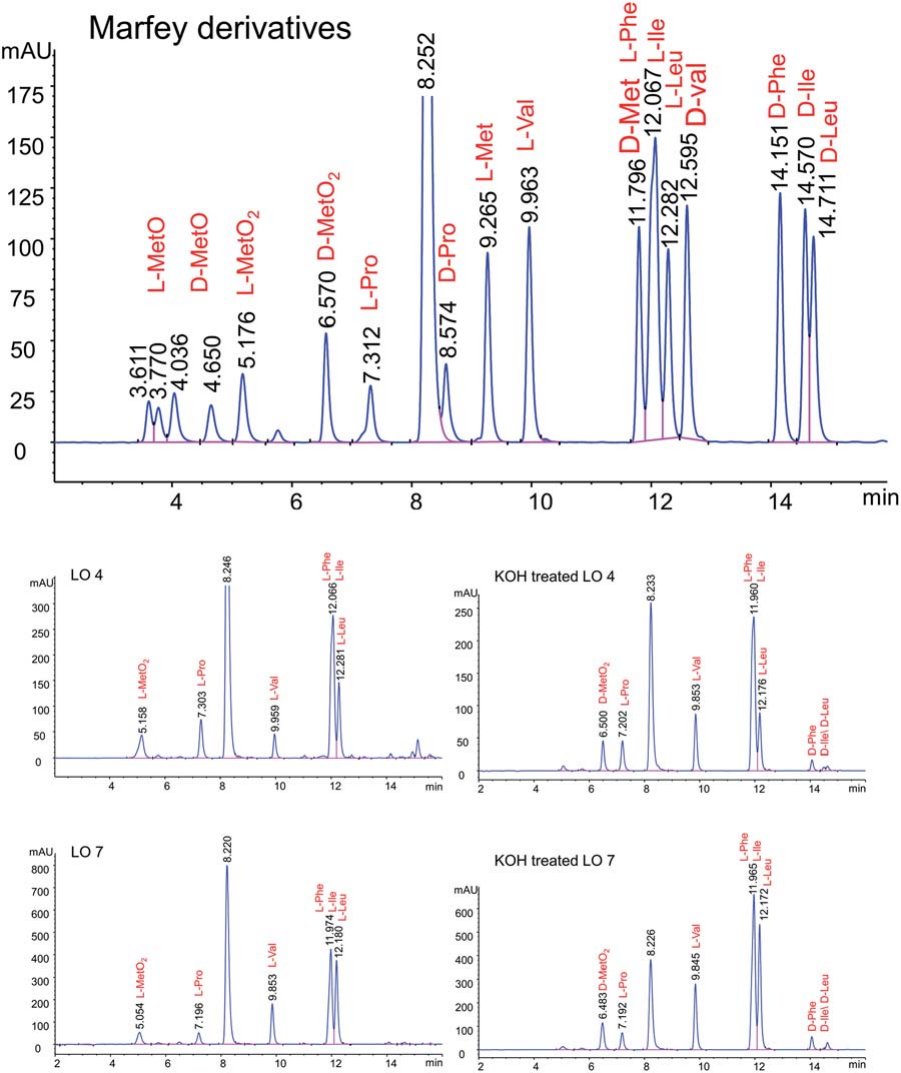
Separation of Marfey derivatized hydrolysate LO 4, LO 7, KOH treated LO 4 and KOH treated LO 7.

LOs were hydrolyzed by heating in 1 M HCl and derivatizing with Marfey's reagent. The optimized HPLC conditions were developed to separate Marfey's derivatives of 8 amino acids (Met, MetO, MetO_2_, Pro, Val, Phe, Leu, IIe) using a single chromatographic gradient (Fig. 3). l-Amino acid Marfey's derivatives eluted before the corresponding d-isomer. The optimized chromatographic conditions were used to compared hydrolyzed LO 4 and purified KOH treated LO 4 (LO 10) after derivatizing with Marfey's reagent. HPLC analysis showed selective epimerization at α-H of MetO_2_ in LO 10 with the formation of d-MetO_2_. These results confirm that the alkali catalyzed reaction proceeded efficiently to produce the d-isomer product. Similar, results were obtained with KOH treated LO 7 (LO 13) shown in Fig. 3. Small additional peaks indicated that side-reactions occurred where non-Met residues (d-Phe/d-Ile/d-Leu) were also isomerized. Alkaline treatment was also performed on reduced LOs of 4 (2 and 3) and 7 (5 and 6), and the products analyzed. However, in this case other LOs 2, 3, 5 and 6 were not purified after KOH treatment but their isomers were confirmed by comparison with available synthetic d-isomers (ESI, Fig. S12–S20†). It was found that LOs 2, 3, 5, and 6 show racemization of Met and MetO amino acids. KOH-treated LO 2 shows the formation of an additional peak in HPLC with similar MS/MS fragmentation (ESI, Fig. S1†), and KOH-treated LO 5 showed 3 peaks in the chromatogram with same molecular mass. It is difficult to characterize the third peak, it might be proline isomerization (*cis*–*trans*) of d-Met or another amino acid isomerization. We are claiming that Met isomerization is kinetically favored over other amino acids due to its orientation in cyclic peptide/reactivity, though other amino acids might also isomerize (ESI, Fig. S2†). The eight membered ring (LOs 5, 6 and 7) shows a higher epimerization rate in the order of MetO_2_ > MetO > Met residues while in nine membered (LOs 2, 3 and 4) the order was MetO > MetO_2_ > Met (Table 1).

**Fig. 4:**
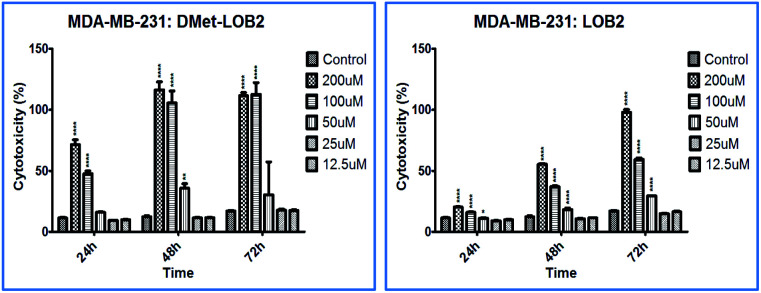
% cytotoxicity of d-Met-LO 8 and l-Met LO 2 against MDA-MB-231 cell lines.

Cytotoxicity studies were designed to understand the effect of isomerization on biological activity. Both natural and synthesized d-analogs were studied using MDA-MB-231 and Sk-Br-3 cell lines (ESI, Fig. S3–S11†). There were cytotoxicity differences observed between l- and d-pair and a higher concentration of 200 μM was required for full inhibition in these cell lines. MetO pairs show low cytotoxicity and the most notable difference was found in d-Met LO 8 with cytotoxicity of ∼100% at 48 h at 100 μM concentration against the MDA-MB-231 cells compared to ∼40% for l-Met-LO 2 (Fig. 4).

We have developed selective chemistry for isomerizing Met, MetO, and MetO_2_. Hence, this ability followed by modification is a route to more chemical novelty. For example, these Met side chains have been successfully coupled with dye, affinity matrix and protein for use as fluorescent tags, protein purification and antibodies production.^33–35^ Click type chemistry was also developed to alkylate Met to prepare multifunctional polypeptides.^36^ Also, ionic copolypeptide vesicles and sulfonium tethered peptides were also prepared.^37,38^

## 4. Conclusions

In summary, orbitides containing one Met and its oxidized forms produce d-analogs under alkaline conditions by selective epimerization of Met, MetO, and MetO_2_. Both d and l-isomers isomers were isolated, hydrolyzed, and derivatized with Marfey's reagent to confirm the amino acid configuration. The eight membered ring and MetO/MetO_2_ were more prone to epimerization. The selective epimerization on cyclic peptides will help to generate rapid structural analogs for future SAR studies. Cyclic peptide conformational orientation was utilized for selective functionalization and have potential to similar cyclic peptides systems. Cytotoxicity studies show the difference between d and l-isomers at higher concentrations. These orbitides can act as a potential biomarkers for \food processing and biomedical applications.

## Conflicts of interest

There are no conflicts to declare.

## Supplementary Material

RA-011-D1RA04260B-s001
