# Supplementary material for: Methionine epimerization in cyclic peptides
Source: RSC Adv. 2021 Jun 11;11(34):20859–64. doi: 10.1039/d1ra04260b (PMC9034004; doi:10.1039/d1ra04260b)
Supplement: RA-011-D1RA04260B-s001 [file RA-011-D1RA04260B-s001.pdf]

Electronic Supplementary Material (ESI) for RSC Advances.
This journal is © The Royal Society of Chemistry 2021

Methionine epimerization in cyclic peptides

Pramodkumar D. Jadhav[a,*], Jianheng Shen[a], Peta-Gaye Burnett[a], Jian Yang[b], Ramaswami Sammynaiken[c], Martin J.T. Reaney[a,d,e,*]

[a] Department of Plant Sciences, University of Saskatchewan, Saskatoon, SK S7N 5A8, Canada

[b] Drug Discovery and Development Research Group, College of Pharmacy and Nutrition, University of Saskatchewan, 107 Wiggins Road, Saskatoon, SK S7N 5E5, Canada

[c]Saskatchewan Structural Sciences Centre, University of Saskatchewan, 110 Science Place, Saskatoon, SK S7N 5C9, Canada

[d]Prairie Tide Diversified Inc., 102 Melville Street, Saskatoon, Saskatchewan S7J 0R1, Canada

[e]Guangdong Saskatchewan Oilseed Joint Laboratory, Department of Food Science and Engineering, Jinan University, 601, Huangpu Avenue West, Guangzhou, Guangdong 510632, China

*Corresponding authors: Tel.: +1 306 966 5027; Fax: +1 306 966 5015.

E-mail address: pramodkumar.jadhav@usask.ca; martin.reaney@usask.ca

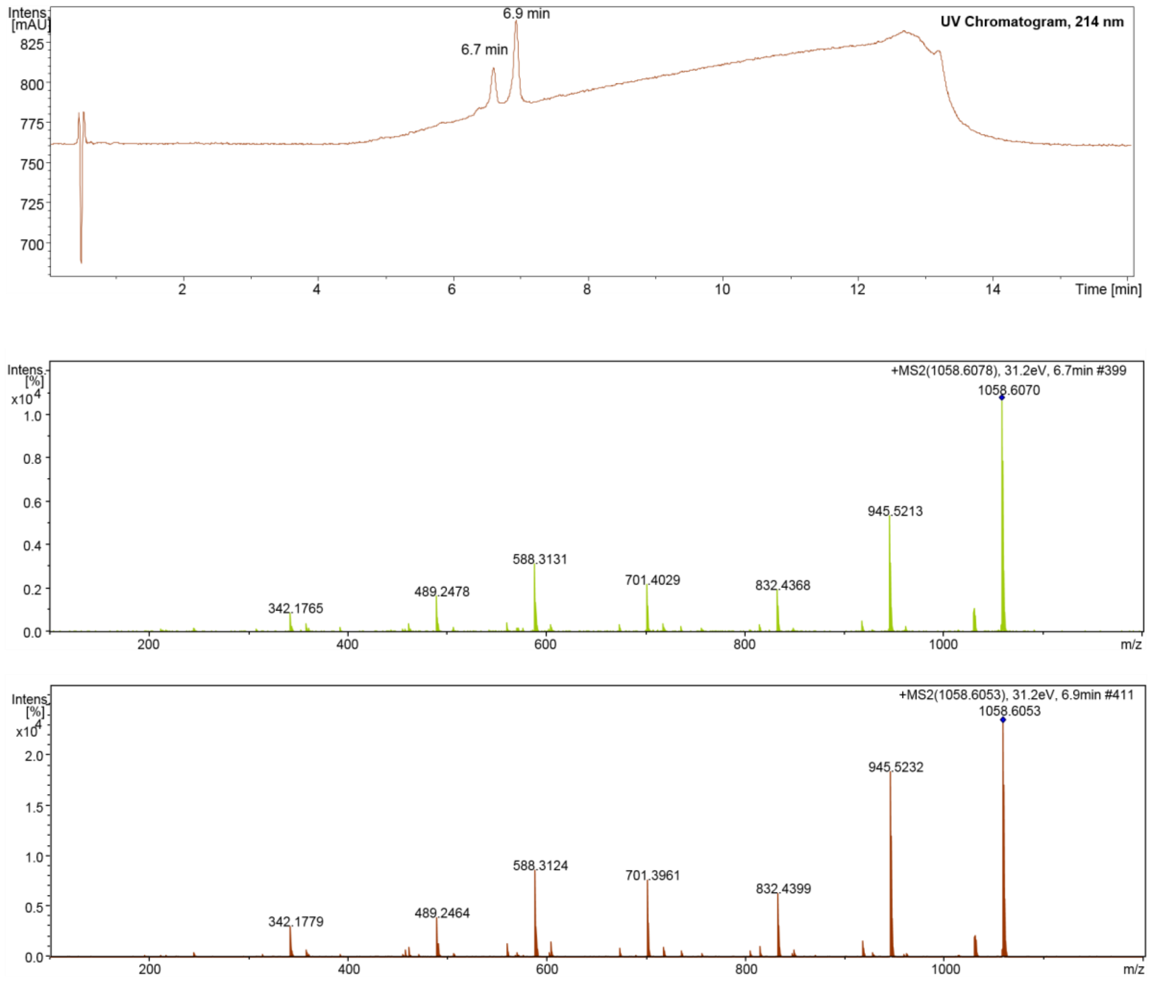

Fig. S1: UV chromatogram of KOH treated LO **2**, MS/MS chromatogram of peak 1 (6.7 min) and peak 2 (6.9 min).

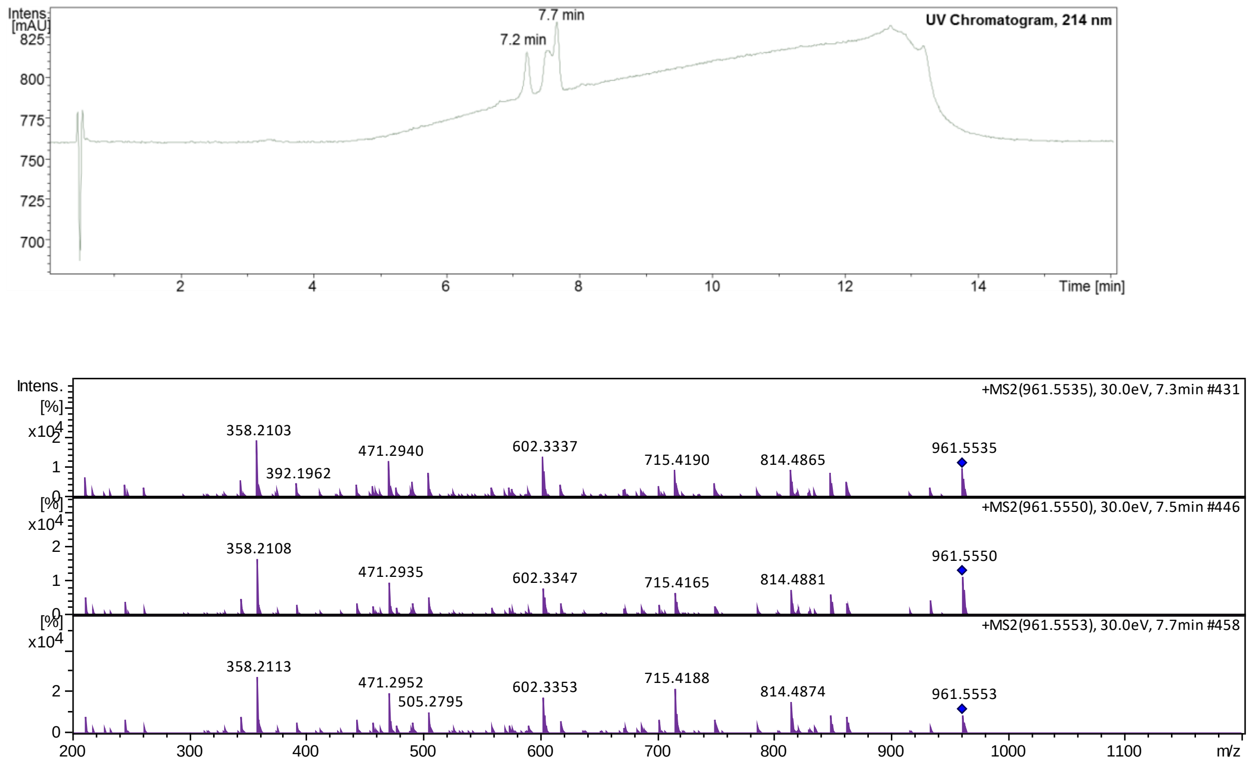

Fig. S2: UV chromatogram of KOH treated LO **5**, MS/MS chromatogram of peak 1 (7.2 min), peak 2 (7.5 min) and peak 3 (7.7 min).

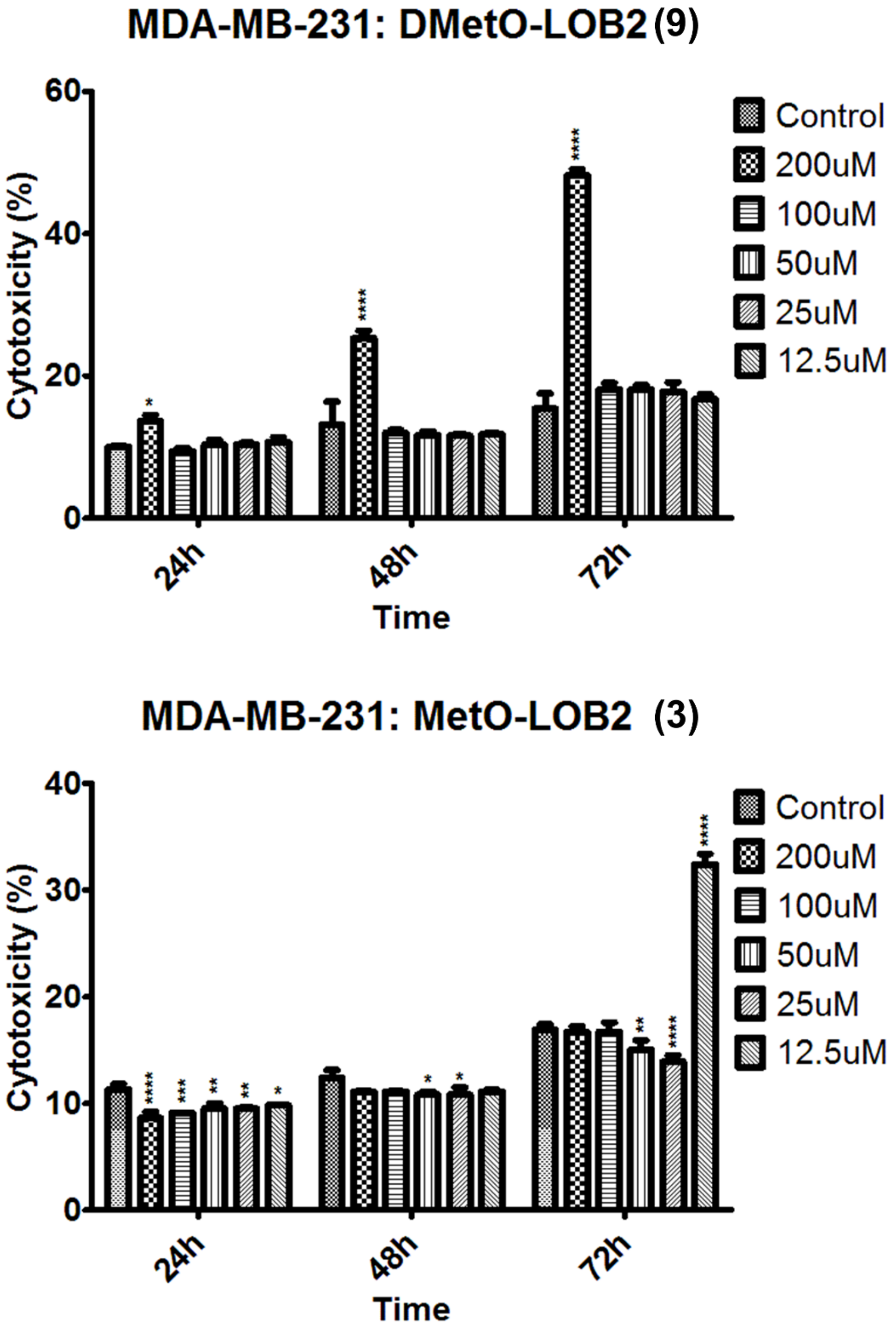

Fig. S3 % cytotoxicity of D-MetO-LOB2 (**9**) and L-MetO-LOB2 (**3**) against the MDA-MB-231 cells.

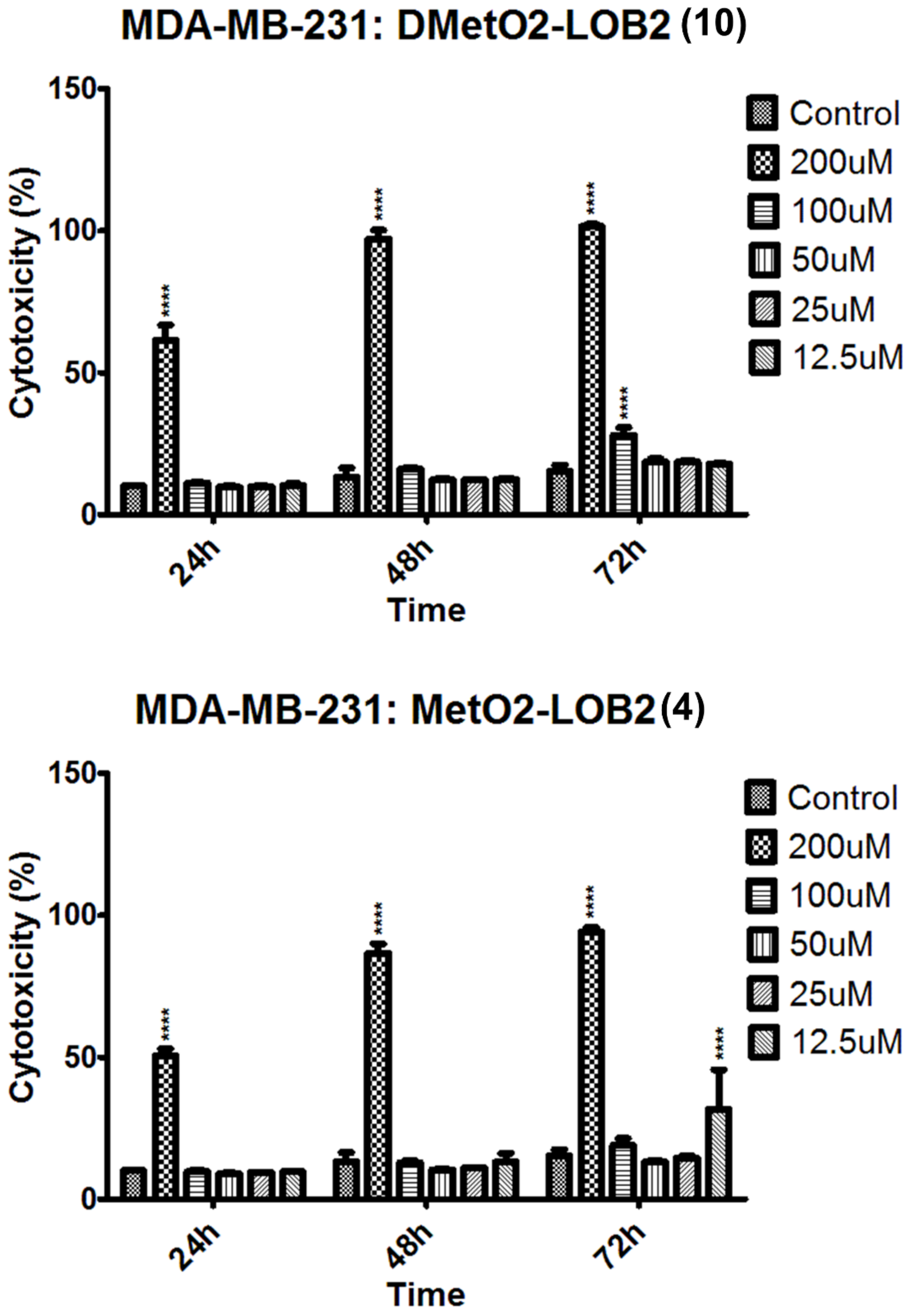

Fig. S4. % cytotoxicity of D-MetO$_2$-LOB2 (**10**) and L-MetO$_2$-LOB2 (**4**) against the MDA-MB-231 cells.

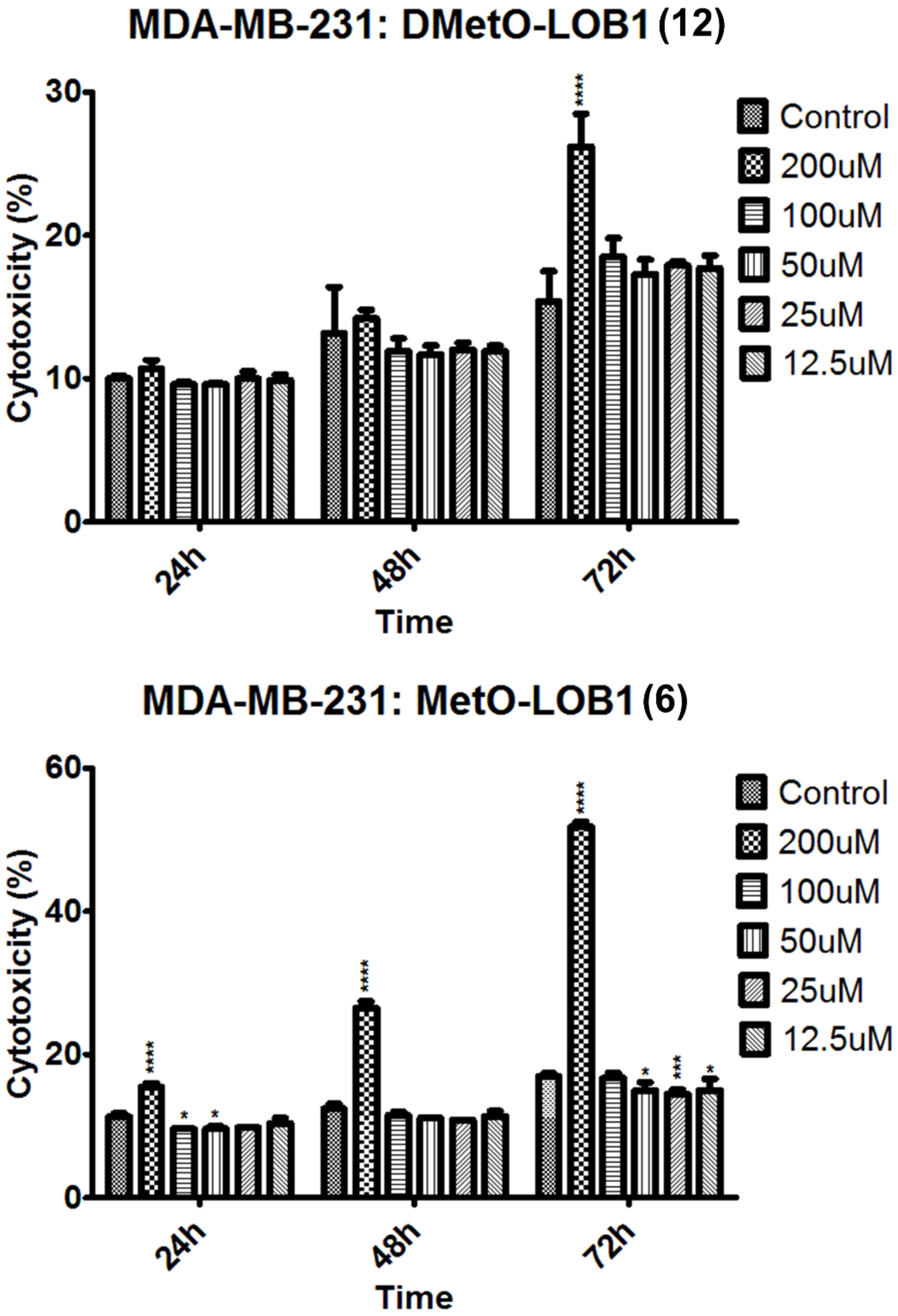

Fig. S5. % cytotoxicity of D-MetO-LOB1 (**12**) and L-MetO-LOB1 (**6**) against the MDA-MB-231 cells.

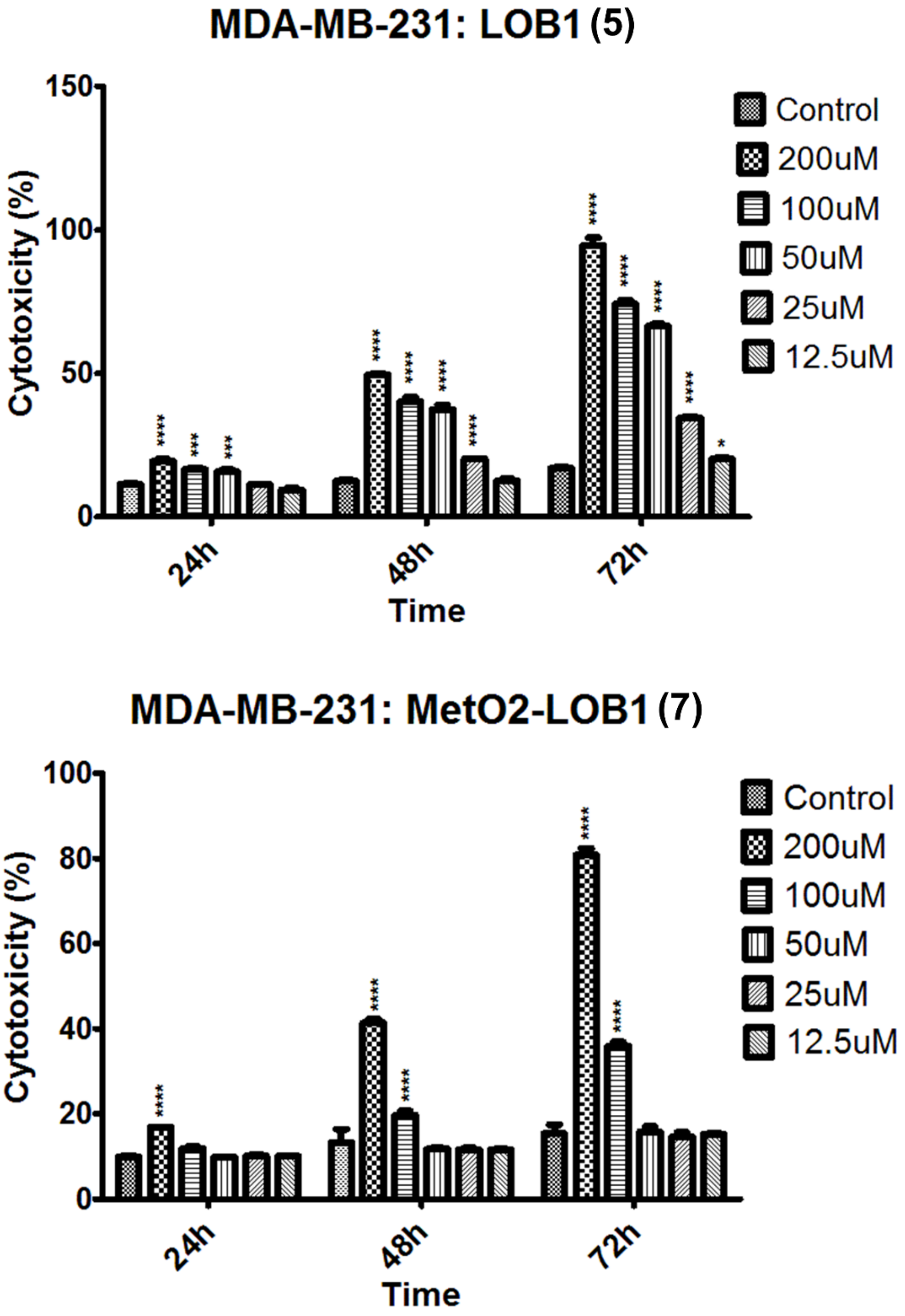

Fig. S6. % cytotoxicity of LOB1 (**5**) and L-MetO$_2$-LOB1 (**7**) against the MDA-MB-231 cells.

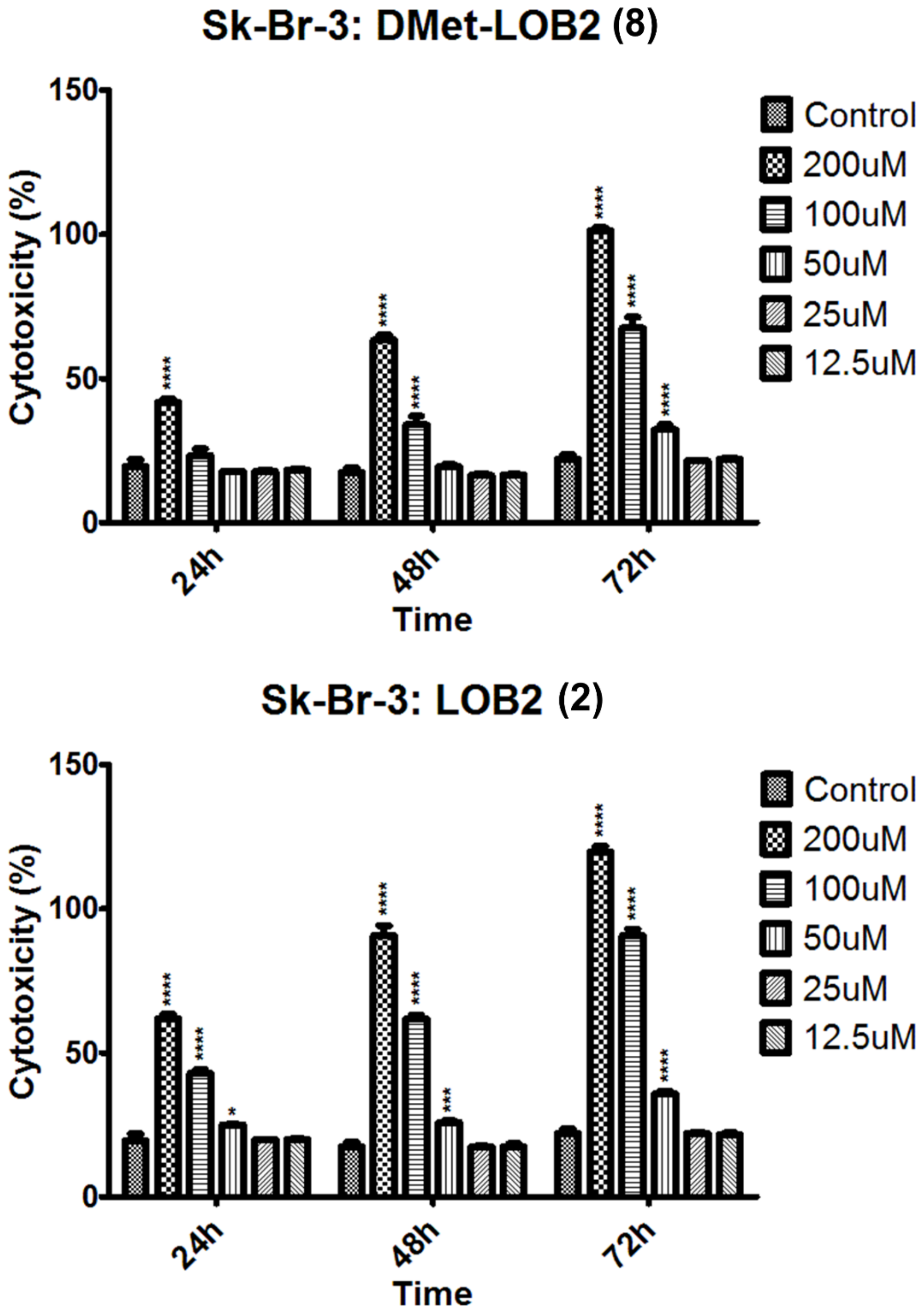

Fig. S7. % cytotoxicity of D-Met-LOB2 (**8**) and LOB2 (**2**) against the Sk-Br-3 cells.

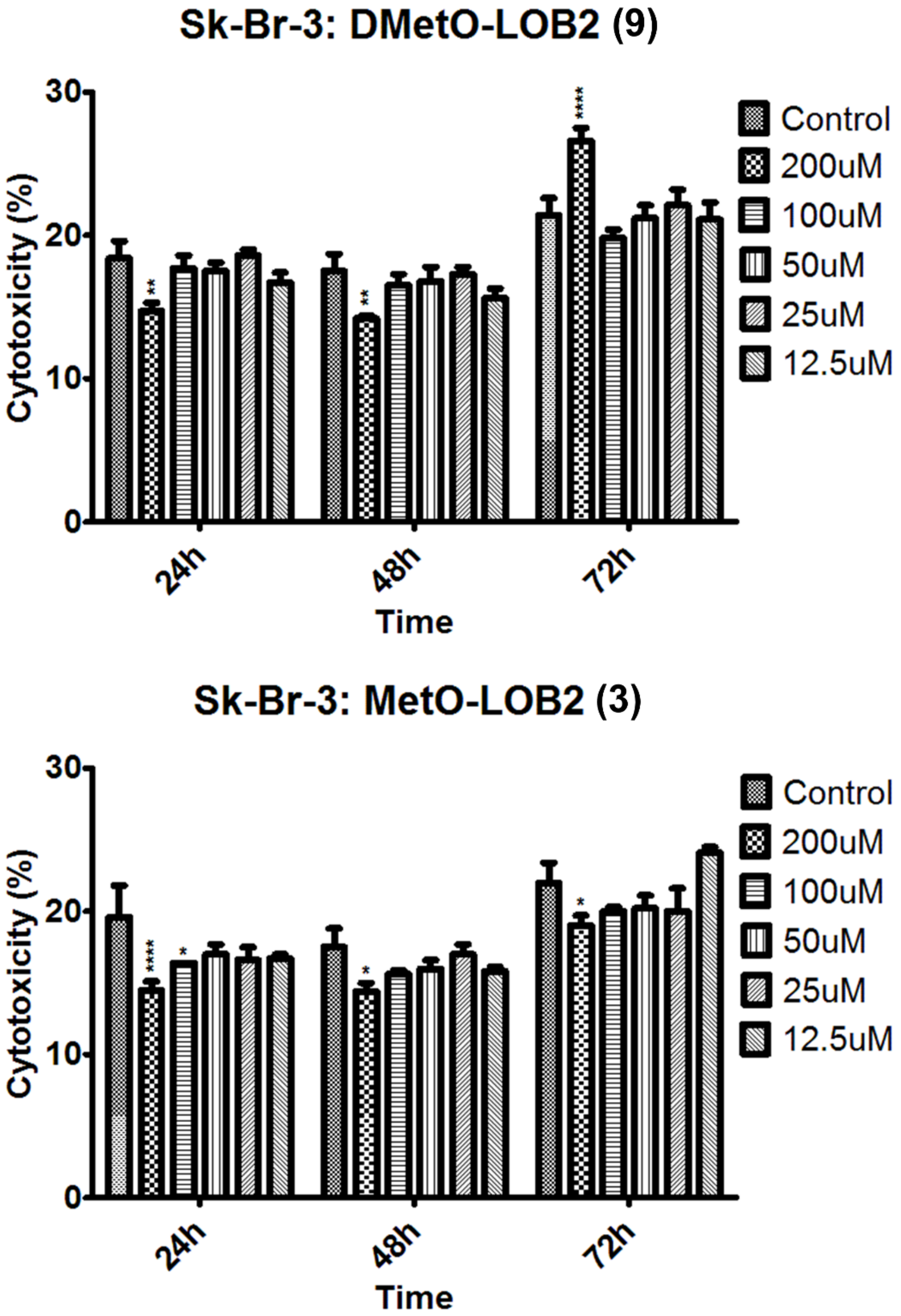

Fig. S8. % cytotoxicity of D-MetO-LOB2 (**9**) and L-MetO-LOB2 (**3**) against the Sk-Br-3 cells.

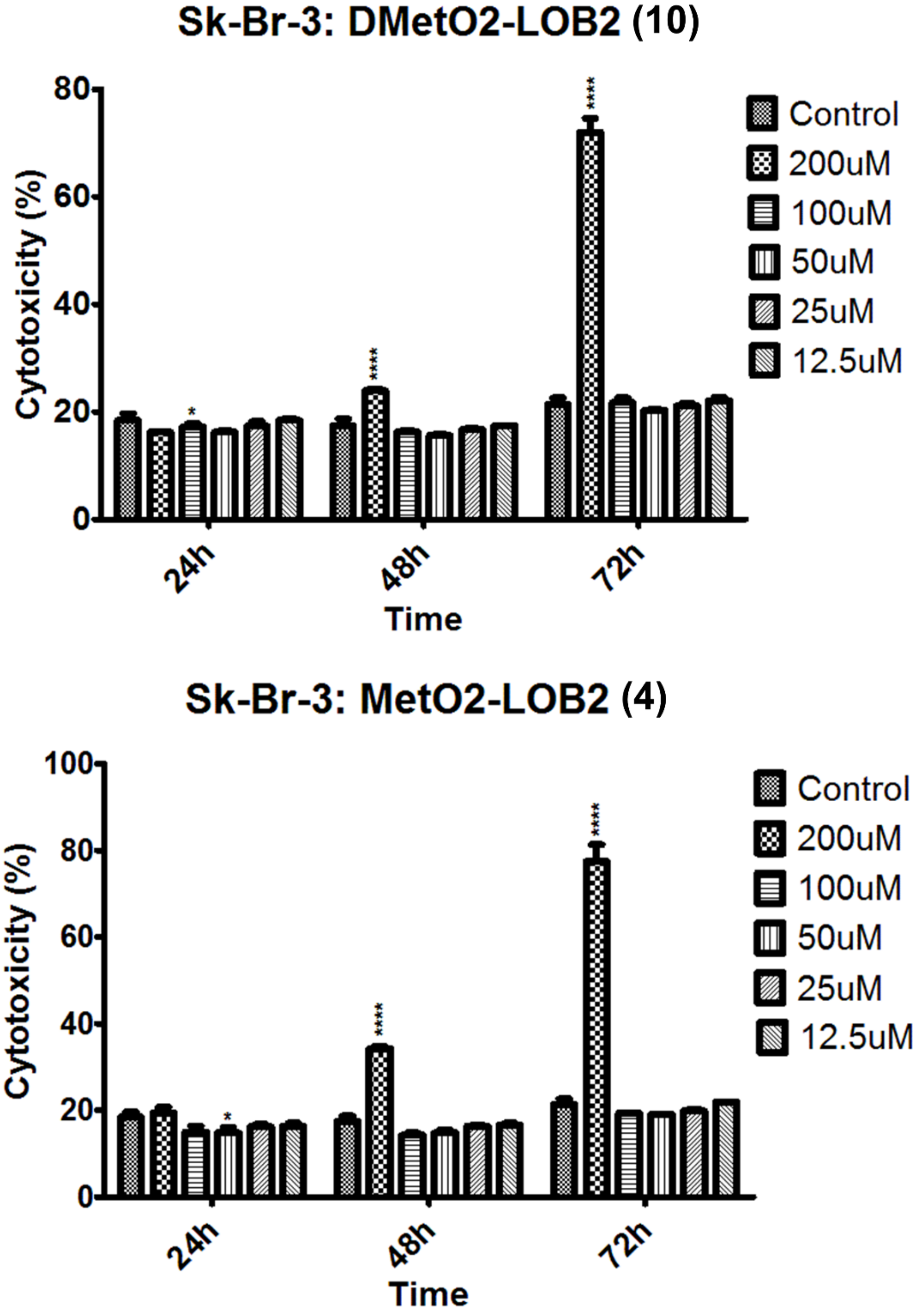

Fig. S9. % cytotoxicity of D-MetO$_2$-LOB2 (**10**) and L-MetO$_2$-LOB2 (**4**) against the Sk-Br-3 cells.

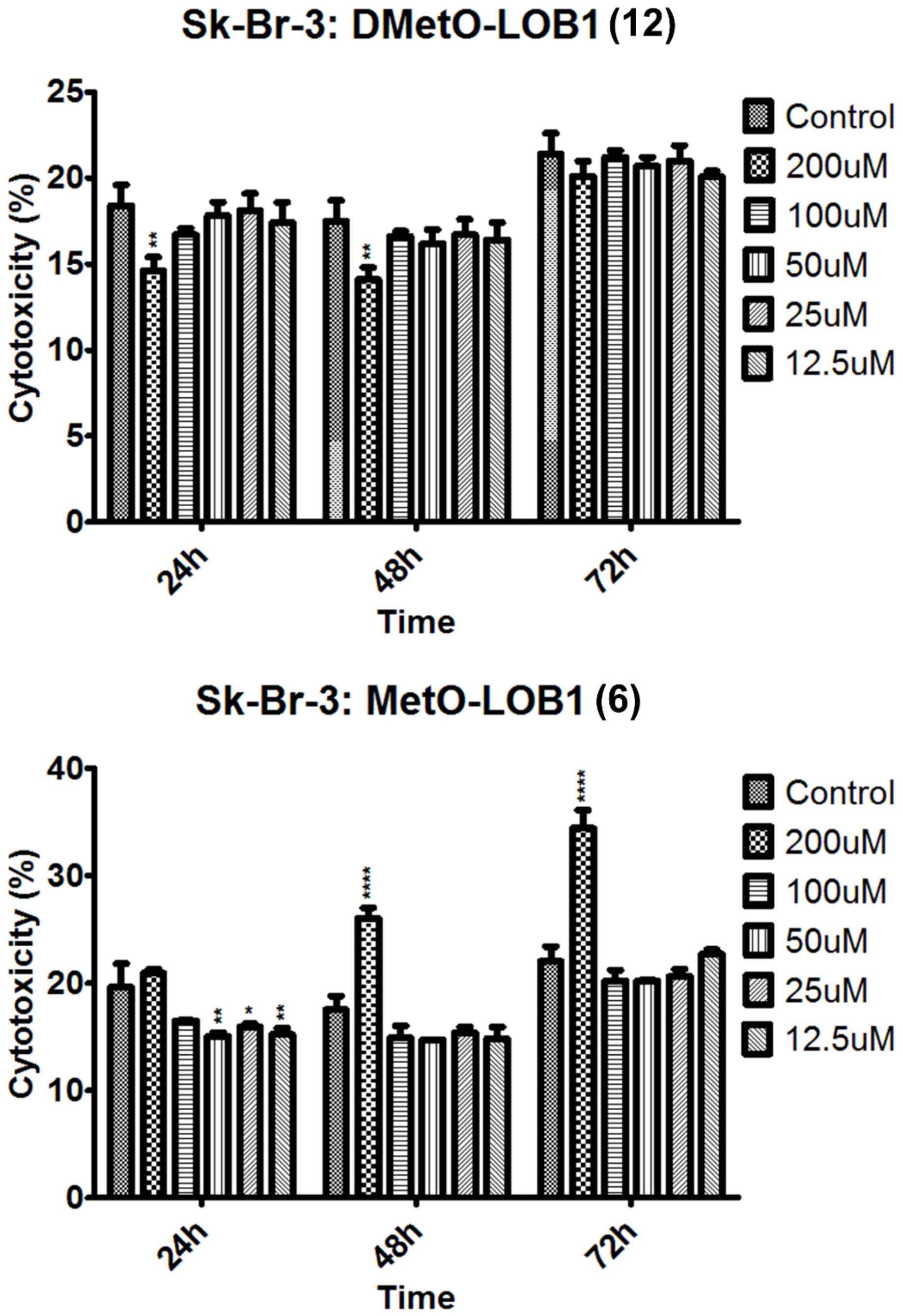

Fig. S10. % cytotoxicity of D-MetO-LOB1 (**12**) and L-MetO-LOB1 (**6**) against the Sk-Br-3 cells.

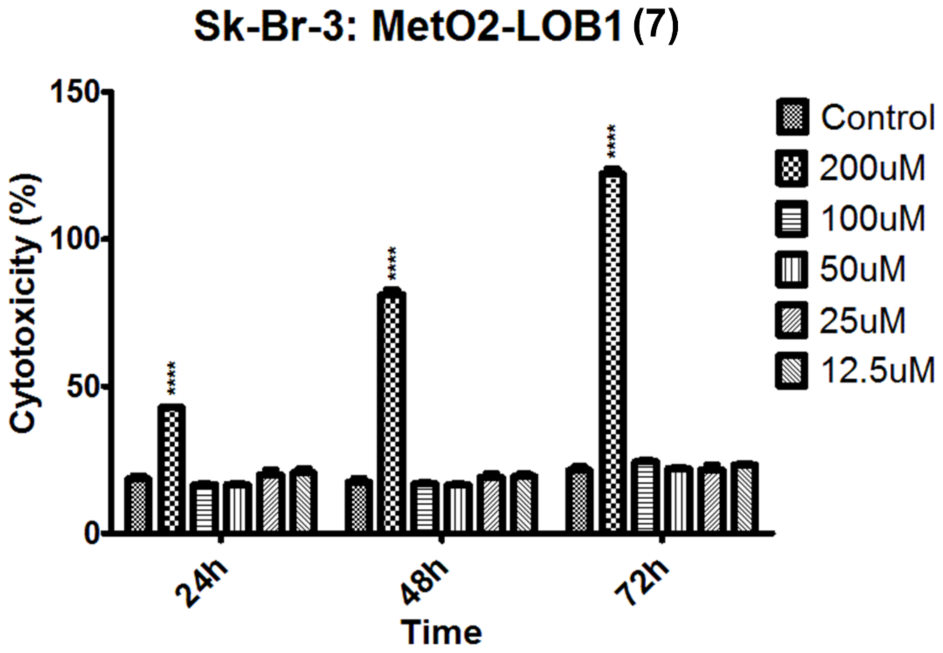

Fig. S11. % cytotoxicity of L-MetO$_2$-LOB1 (**7**) against the Sk-Br-3 cells.

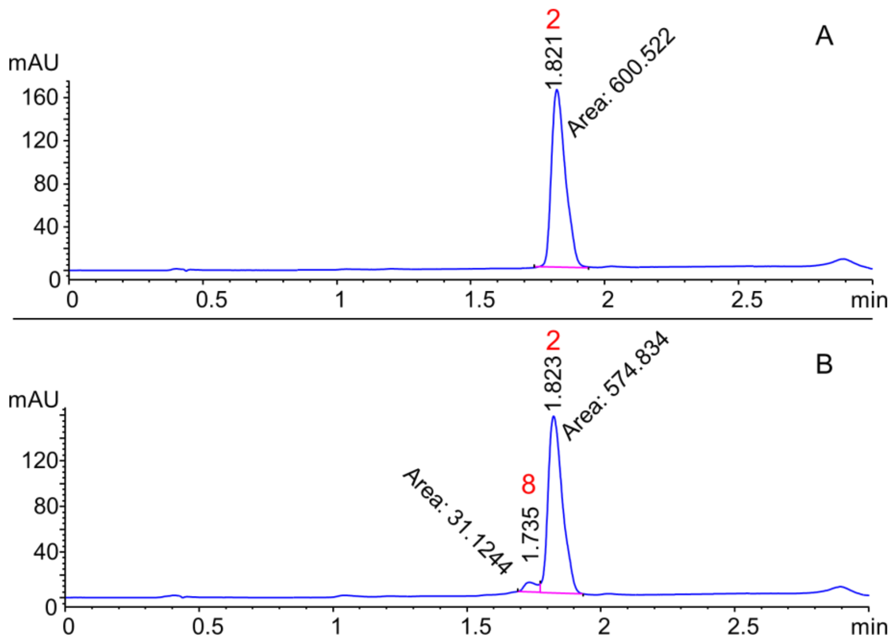

Fig. S12. HPLC chromatogram of (A) LO **2**; (B) KOH treated LO **2**.

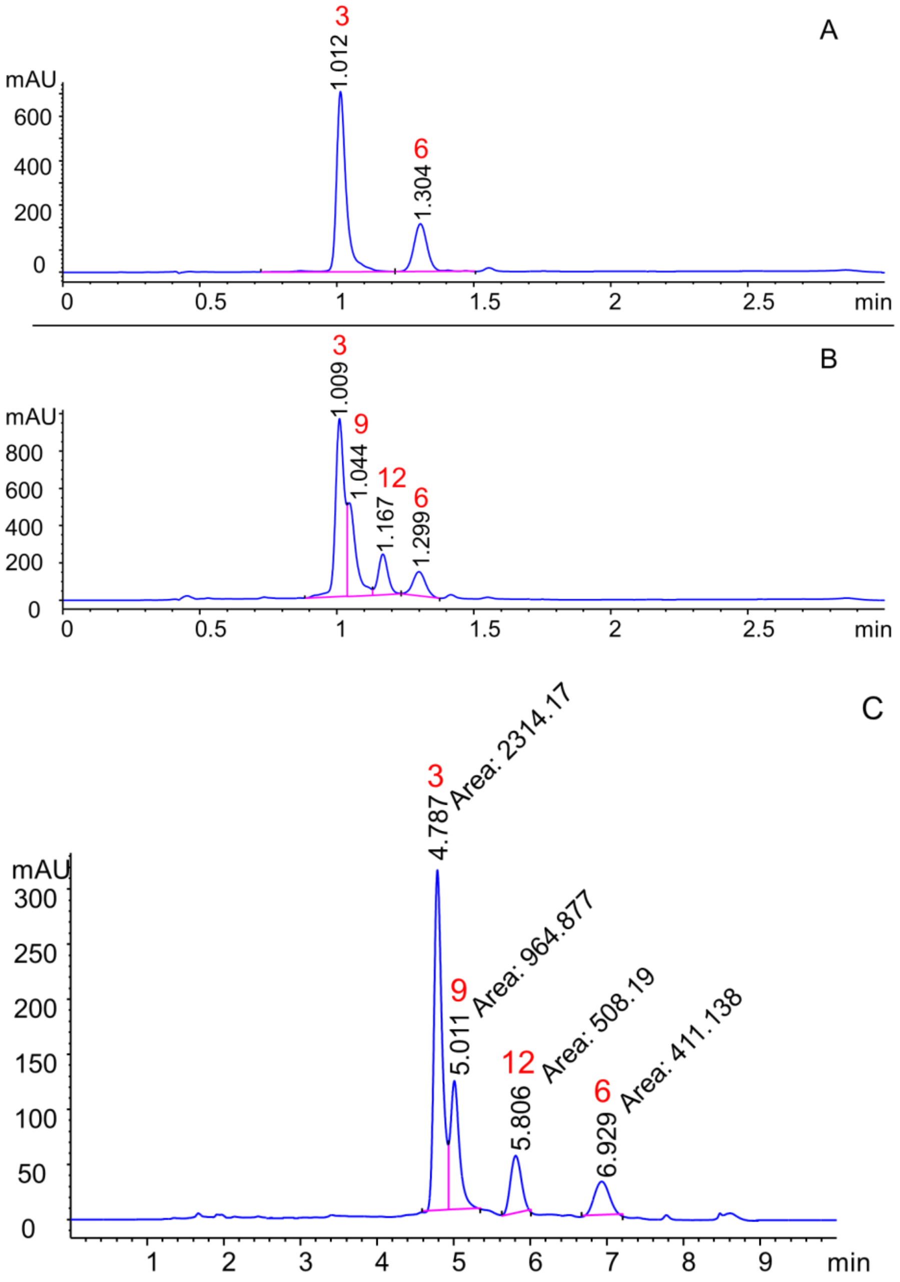

Fig. S13. HPLC chromatogram of (A) LOs **3** and **6**; (B) KOH treated LOs **3** and **6**; (C) KOH treated LOs **3** and **6** (preparative).

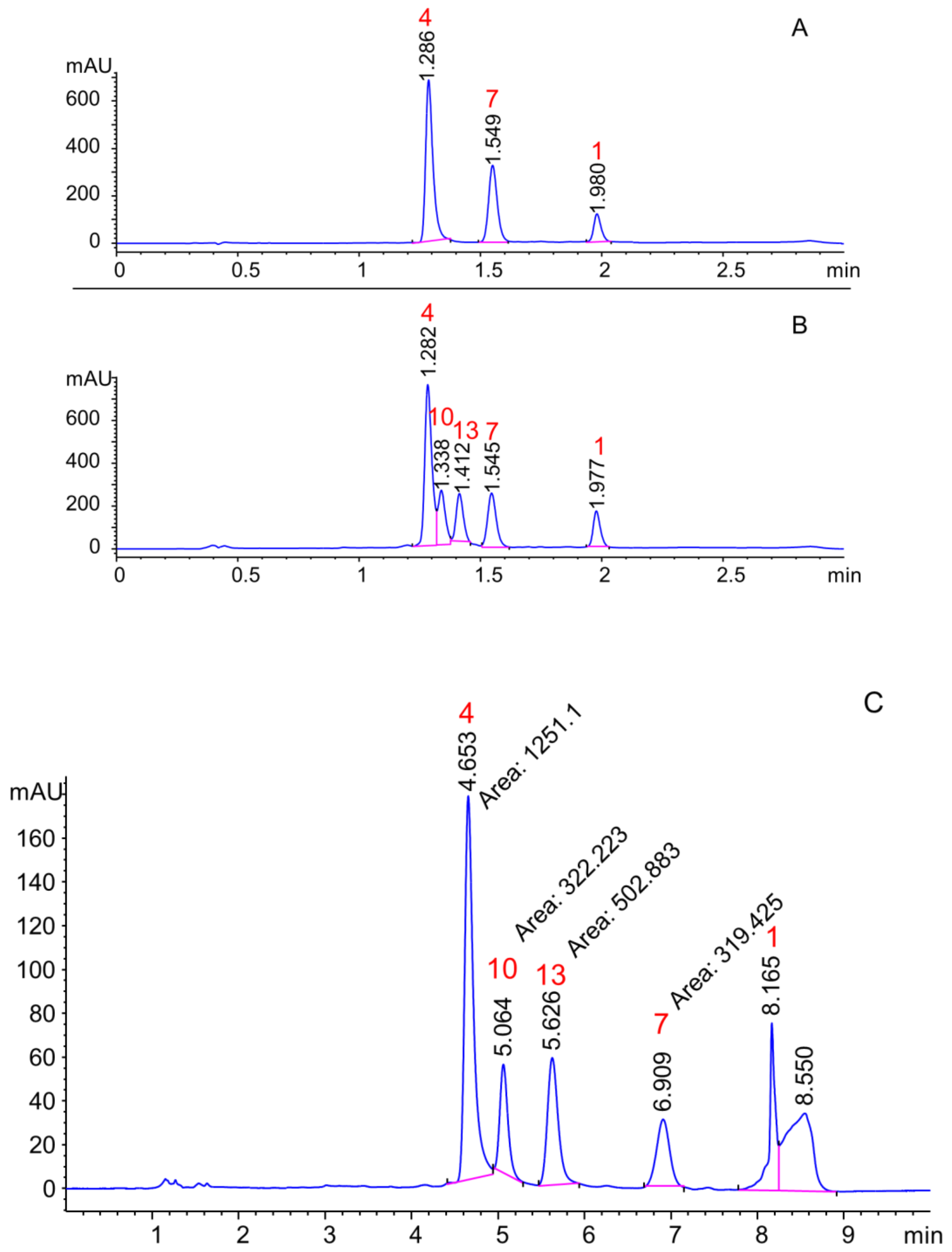

Fig. S14. HPLC chromatogram of (A) LOs **1**, **4** and **7**; (B) KOH treated LOs **1**, **4** and **7**; (C) KOH treated LOs **1**, **4** and **7** (preparative).

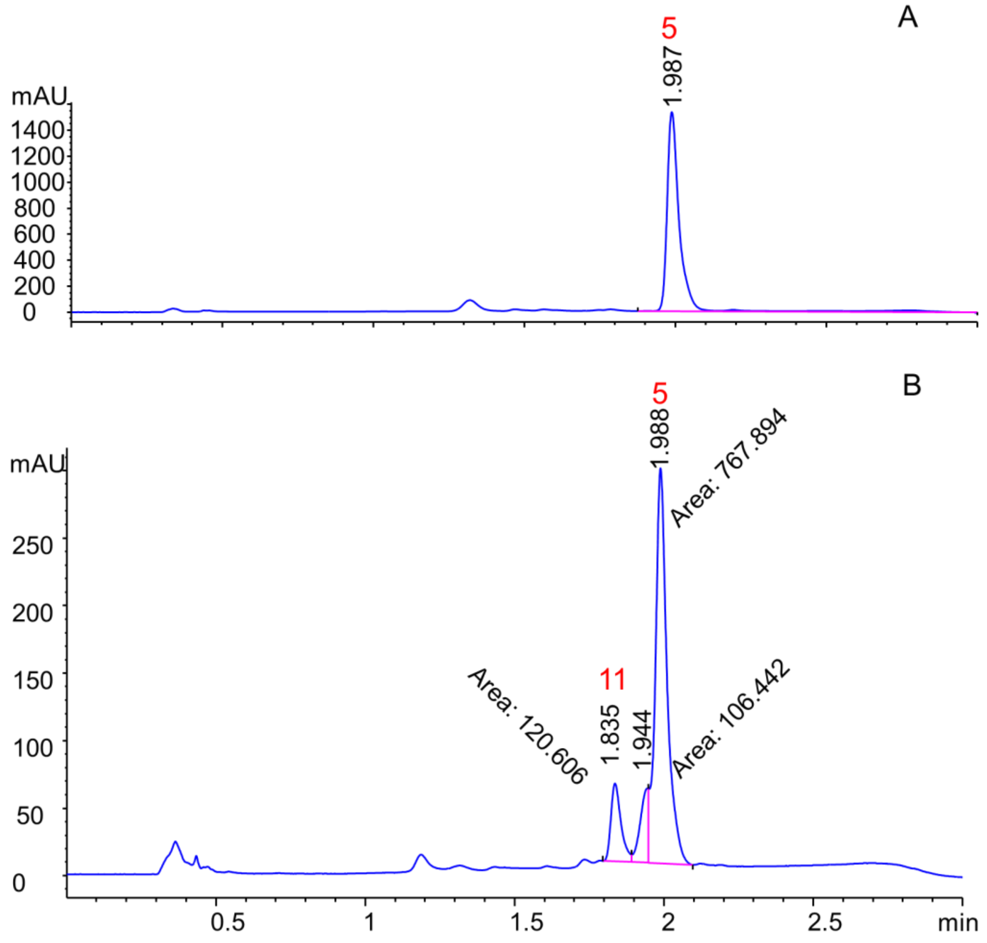

Fig. S15. HPLC chromatogram of (A) LO **5**; (B) KOH treated LO **5**.

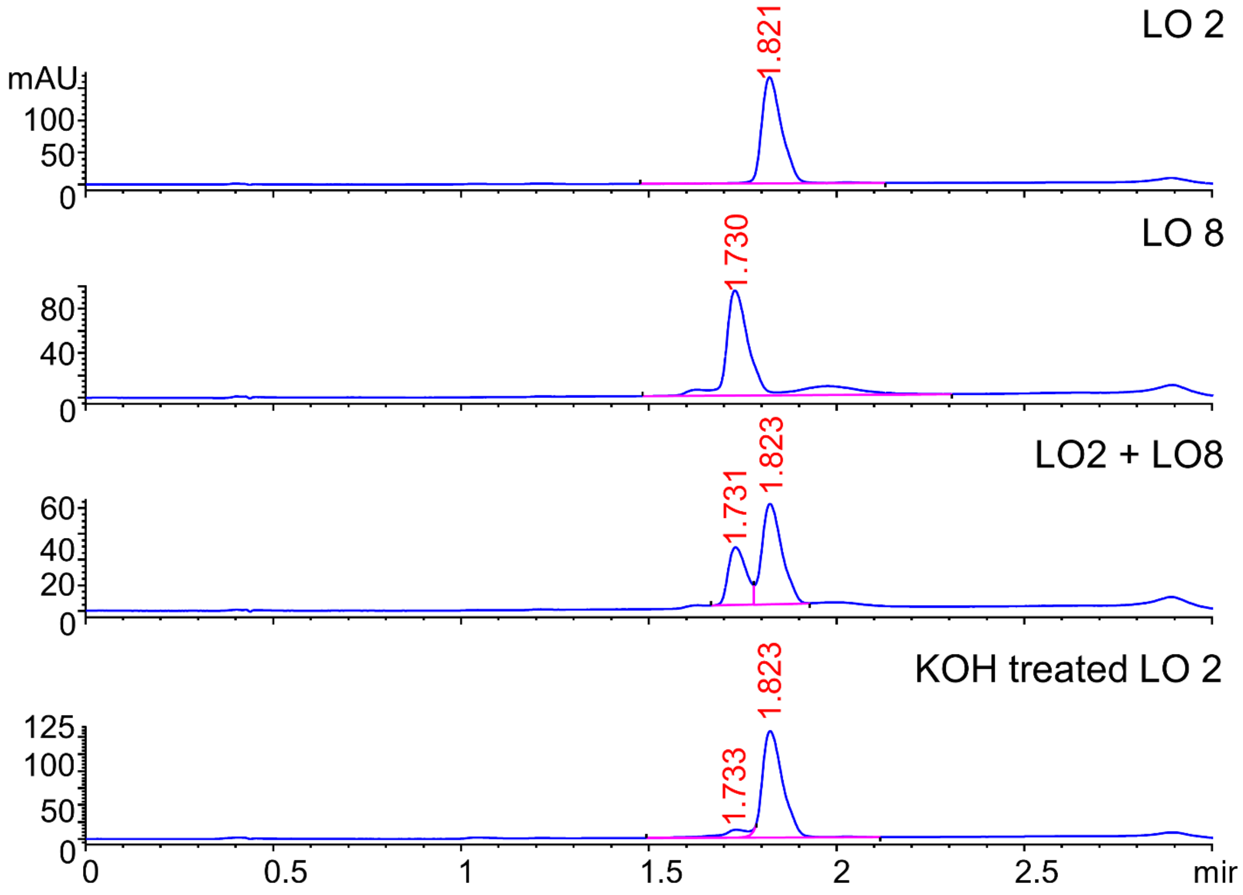

Fig. S16. HPLC chromatogram of LO **2**, LO **8**, LO **2** + LO **8** and KOH treated LO **2**.

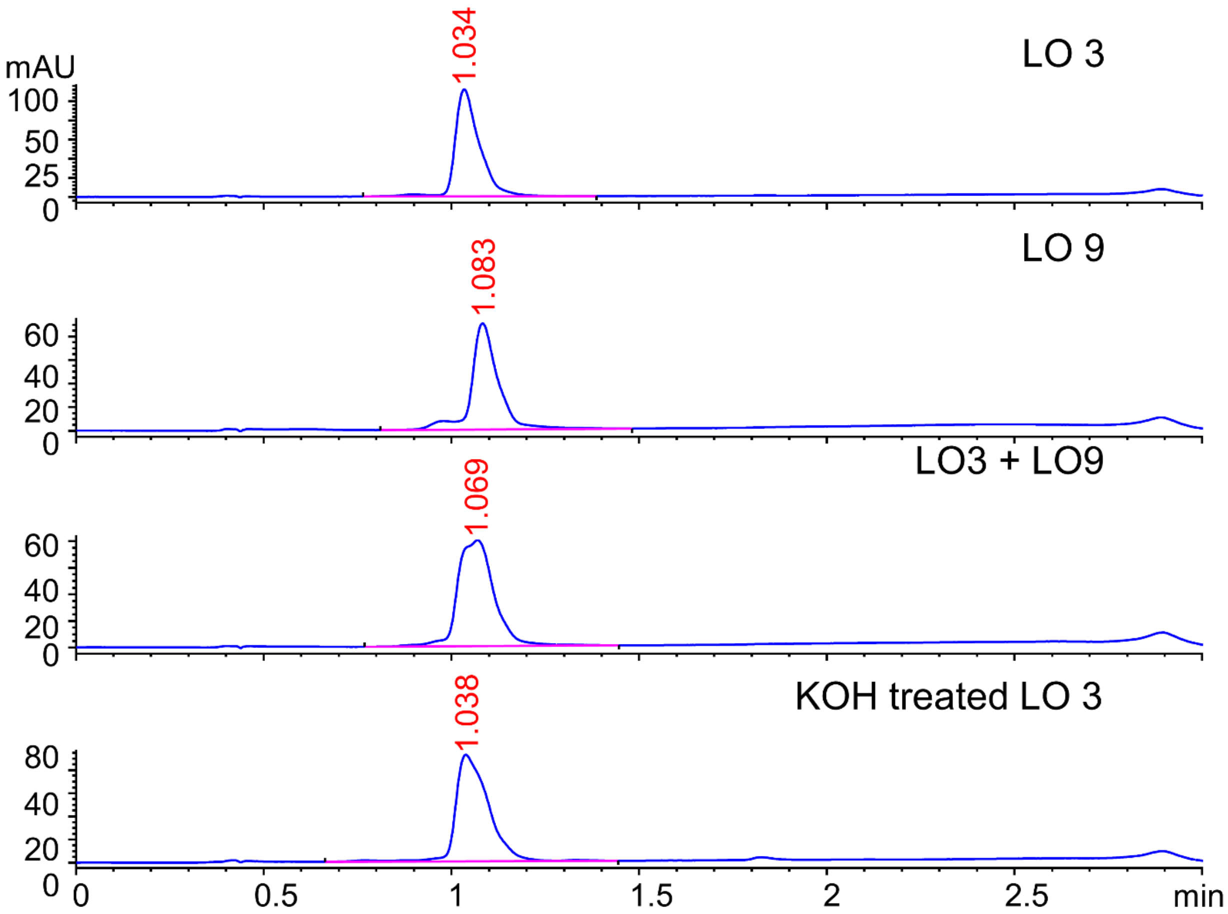

Fig. S17. HPLC chromatogram of LO **3**, LO **9**, LO **3** + LO **9** and KOH treated LO **3**.

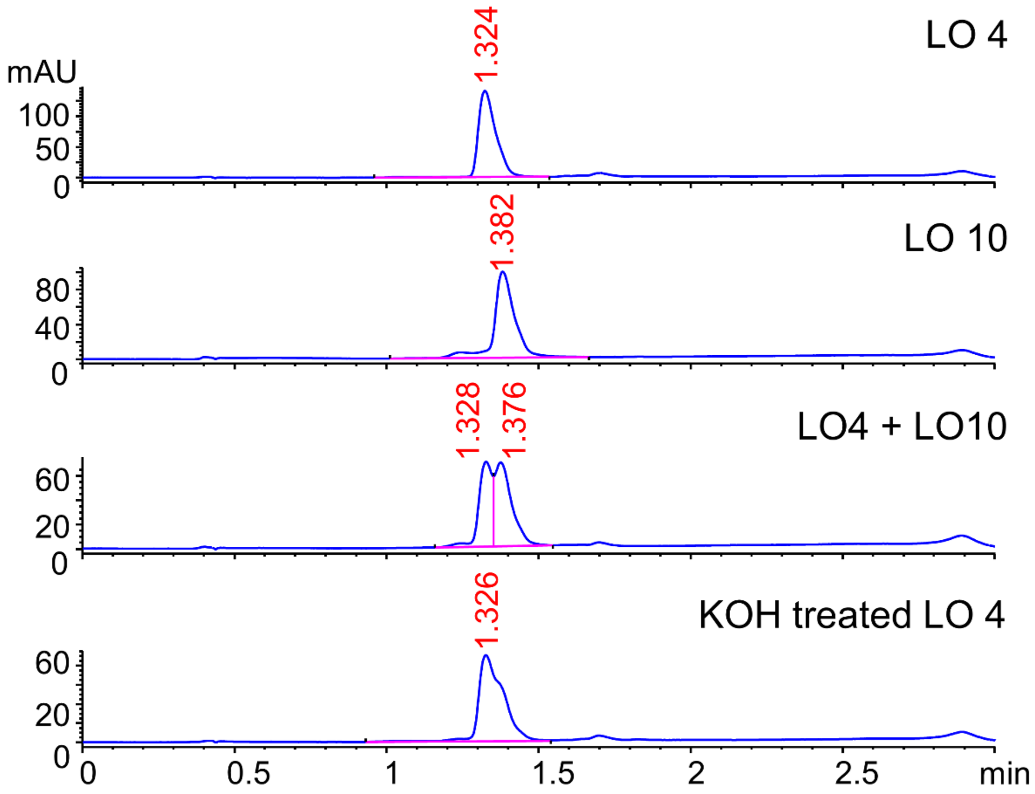

Fig. S18. HPLC chromatogram of LO **4**, LO **10**, LO **4** + LO **10** and KOH treated LO **4**.

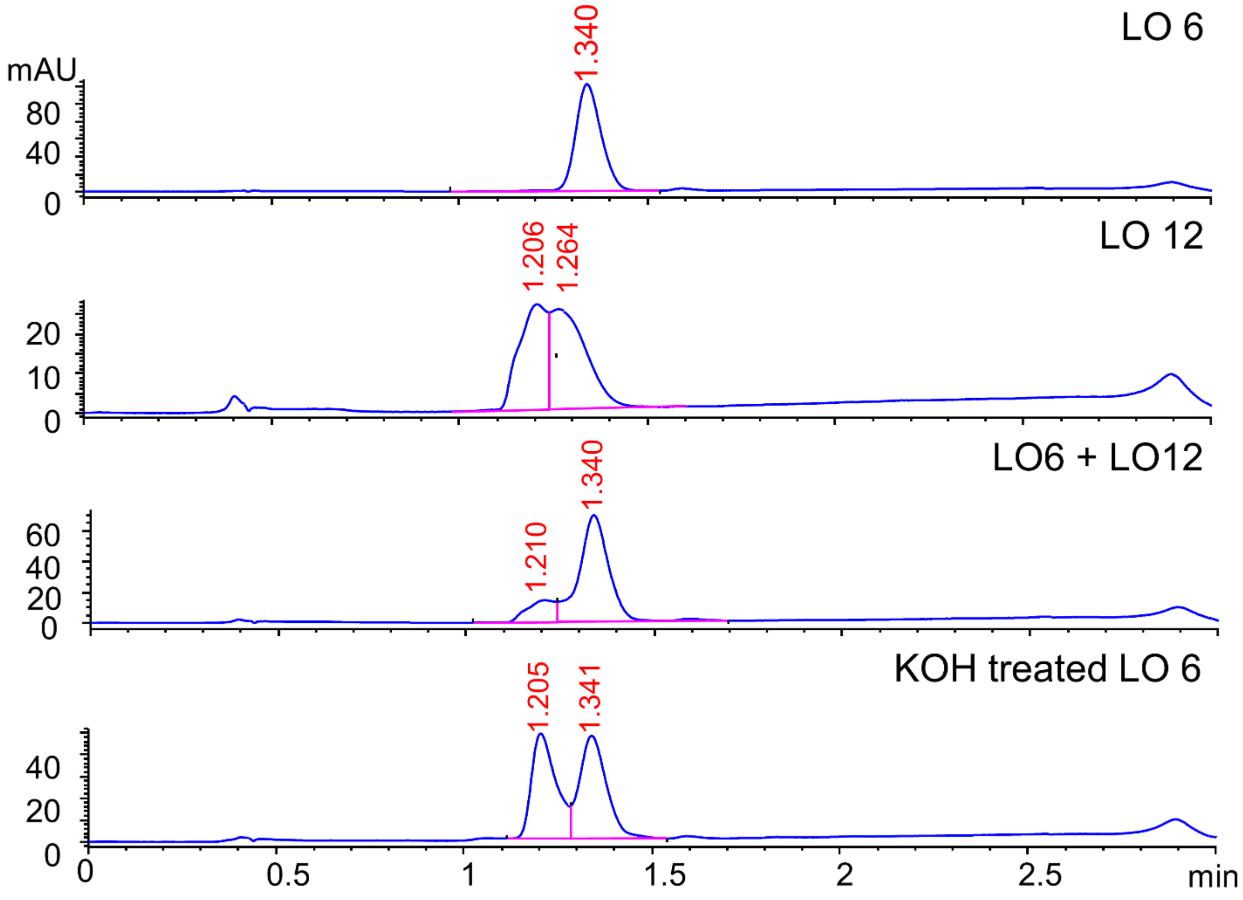

Fig. S19. HPLC chromatogram of LO **6**, LO **12**, LO **6** + LO **12** and KOH treated LO **6**.

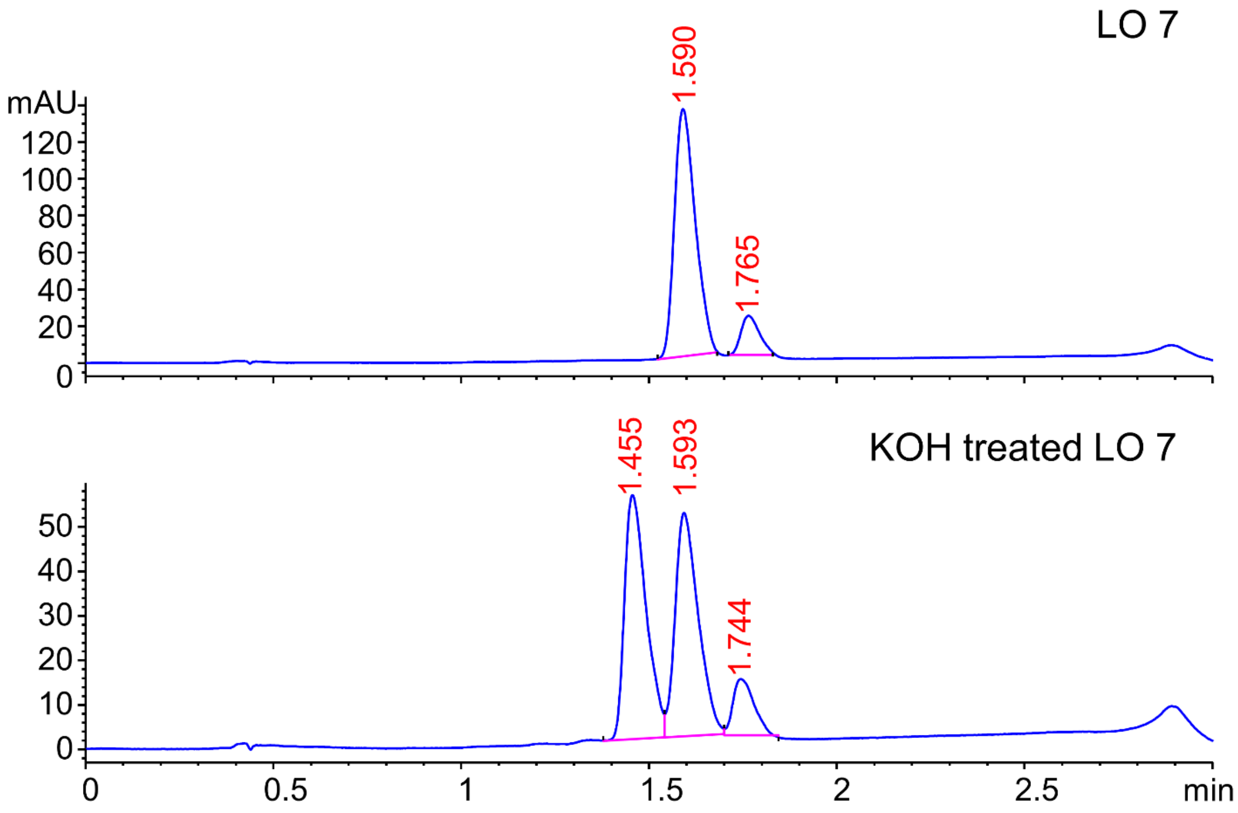

Fig. S20. HPLC chromatogram of LO **7** and KOH treated LO **7**.

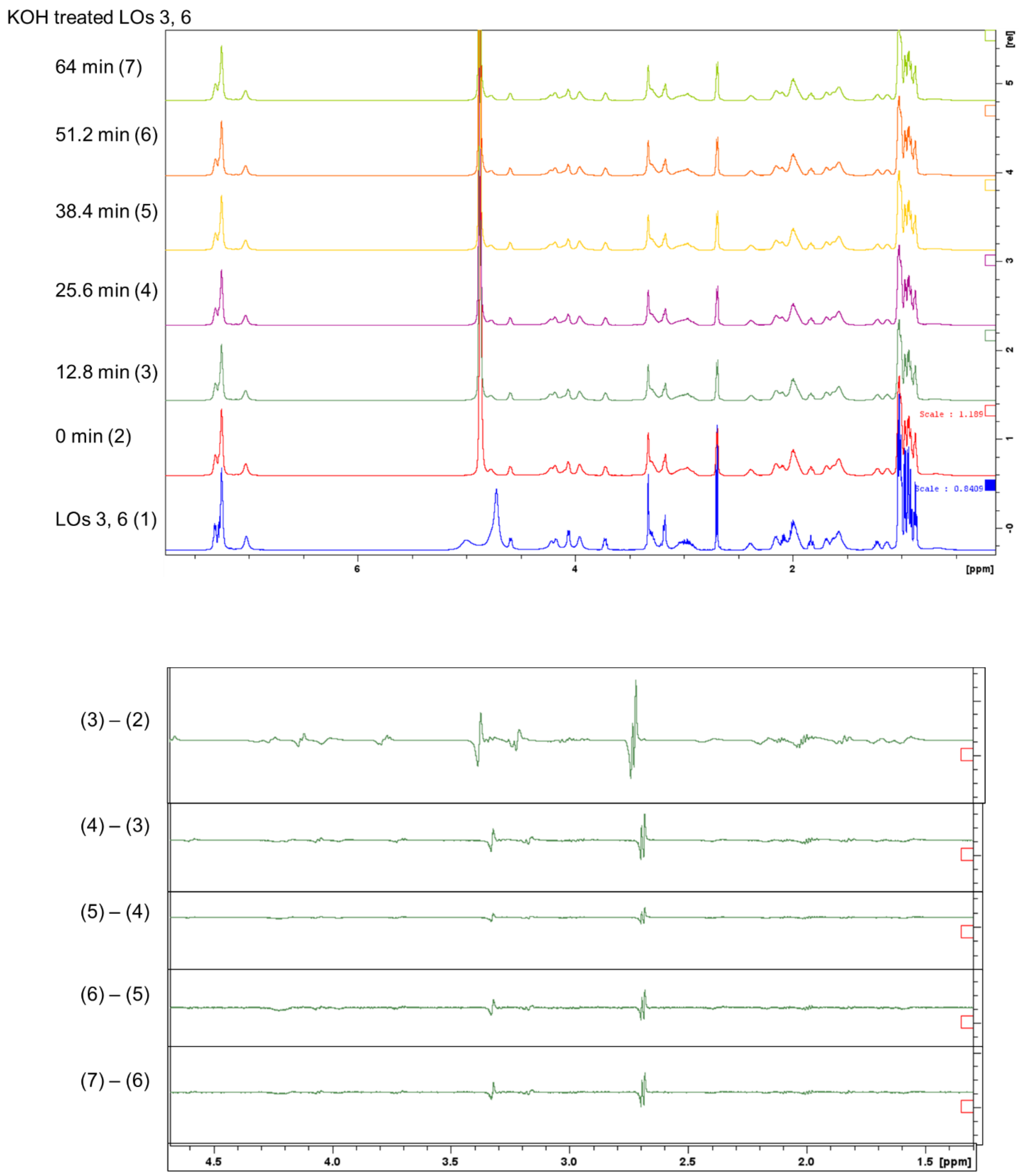

Fig. S21. (A) NMR spectra of LOs **3** and **6** mixture and KOH treated at different time intervals. (B) NMR difference spectra of KOH treated LOs **3** and **6**.

Table S1 Amino acid sequences and masses of LOs **1-13**

LO[a] (code)	amino acid sequence (NαC-)	chemical formula	MW (Da)
[1−9-NαC]-linusorb B3 (**1**)	Ile-Leu-Val-Pro-Pro-Phe-Phe-Leu-Ile	$C_{57}H_{85}N_9O_9$	1040.34
[1−9-NαC]-linusorb B2 (**2**)	Met-Leu-Ile-Pro-Pro-Phe-Phe-Val-Ile	$C_{56}H_{83}N_9O_9S$	1058.38
[1−9-NαC],[1-(R_s,S_s)-MetO]-linusorb B2 (**3**)	[(R_s,S_s)-MetO]-Leu-Ile-Pro-Pro-Phe-Phe-Val-Ile	$C_{56}H_{83}N_9O_{10}S$	1074.38
[1−9-NαC],[1-MetO$_2$]-linusorb B2 (**4**)	MetO$_2$-Leu-Ile-Pro-Pro-Phe-Phe-Val-Ile	$C_{56}H_{83}N_9O_{11}S$	1090.38
[1−8-NαC]-linusorb B1 (**5**)	Met-Leu-Val-Phe-Pro-Leu-Phe-Ile	$C_{51}H_{76}N_8O_8S$	961.26
[1−8-NαC],[1-(R_s,S_s)-MetO]-linusorb B1 (**6**)	[(R_s,S_s)-MetO]-Leu-Val-Phe-Pro-Leu-Phe-Ile	$C_{51}H_{76}N_8O_9S$	977.26
[1−8-NαC],[1-MetO$_2$]-linusorb B1 (**7**)	MetO$_2$-Leu-Val-Phe-Pro-Leu-Phe-Ile	$C_{51}H_{76}N_8O_{10}S$	993.26
[1−9-NαC], DMet-linusorb B2 (**8**)	DMet-Leu-Ile-Pro-Pro-Phe-Phe-Val-Ile	$C_{56}H_{83}N_9O_9S$	1058.38
[1−9-NαC],[1-(R_s,S_s)-DMetO]-linusorb B2 (**9**)	[(R_s,S_s)-DMetO]-Leu-Ile-Pro-Pro-Phe-Phe-Val-Ile	$C_{56}H_{83}N_9O_{10}S$	1074.38
[1−9-NαC],[1-DMetO$_2$]-linusorb B2 (**10**)	DMetO$_2$-Leu-Ile-Pro-Pro-Phe-Phe-Val-Ile	$C_{56}H_{83}N_9O_{11}S$	1090.38
[1−8-NαC], DMet-linusorb B1(**11**)	DMet-Leu-Val-Phe-Pro-Leu-Phe-Ile	$C_{51}H_{76}N_8O_8S$	961.26
[1−8-NαC],[1-(R_s,S_s)-DMetO]-linusorb B1 (**12**)	[(R_s,S_s)-DMetO]-Leu-Val-Phe-Pro-Leu-Phe-Ile	$C_{51}H_{76}N_8O_9S$	977.26
[1−8-NαC],[1-DMetO$_2$]-linusorb B1 (**13**)	DMetO$_2$-Leu-Val-Phe-Pro-Leu-Phe-Ile	$C_{51}H_{76}N_8O_{10}S$	993.26
